# Chloride Channels are Involved in the Development of Atrial Fibrillation – A Transcriptomic and proteomic Study

**DOI:** 10.1038/s41598-017-10590-w

**Published:** 2017-08-31

**Authors:** Yi-Yao Jiang, Hai-Tao Hou, Qin Yang, Xiao-Cheng Liu, Guo-Wei He

**Affiliations:** 1 Department of Cardiovascular Surgery & Center for Basic Medical Research, TEDA International Cardiovascular Hospital, The Chinese Academy of Medical Sciences & Peking Union Medical College, & Nankai University, Tianjin, China; 2 The Affiliated Hospital of Hangzhou Normal University & Zhejiang University, Hangzhou, China; 3 Department of Surgery, Oregon Health and Science University, Portland, Oregon USA

## Abstract

Electrical and structural remodeling processes are contributors to the self-perpetuating nature of atrial fibrillation (AF). However, their correlation has not been clarified. In this study, human atrial tissues from the patients with rheumatic mitral valve disease in either sinus rhythm or persistent AF were analyzed using a combined transcriptomic and proteomic approach. An up-regulation in chloride intracellular channel (CLIC) 1, 4, 5 and a rise in type IV collagen were revealed. Combined with the results from immunohistochemistry and electron microscope analysis, the distribution of type IV collagen and effects of fibrosis on myocyte membrane indicated the possible interaction between CLIC and type IV collagen, confirmed by protein structure prediction and co-immunoprecipitation. These results indicate that CLICs play an important role in the development of atrial fibrillation and that CLICs and structural type IV collagen may interact on each other to promote the development of AF in rheumatic mitral valve disease.

## Introduction

Atrial fibrillation (AF) is the most frequently occurred and sustained atrial arrhythmia. The estimated prevalence of AF in the general population is as high as 1–2%^1^. In patients undergoing mitral valve operations, 60% are affected by AF^2^. AF is associated with significant negative impact on quality of life, morbidity and mortality, and length of hospital stay with increased health care costs^3^.

With electrical and structural atrial remodeling, AF begins in a paroxysmal form, progressing through persistent to permanent^4, 5^. Left atrial (LA) remodeling is a maladaptive process, including fibroblast proliferation, collagen accumulation, myocyte hypertrophy, and apoptosis^6^. Moreover, studies have focused on the mechanism responsible to AF-induced changes in the electrophysiological properties of atrial tissues and ion channels in the cell membrane. In fact, changes in the channels, mainly involving the Ca^2+^, K^+^ and Na^+^ ion channels, in the atrial myocytes have been reported^7–9^.

Chloride channels are ubiquitously expressed, being localized both in plasma membrane and in intracellular organelles^10^. The chloride intracellular channel (CLIC) proteins are highly conserved in vertebrates and successive analysis of human CLIC isoforms demonstrates that nine CLICs have been found in humans^11–16^. Like other ion channels, CLICs function in the plasma membrane or in membranes of intracellular organelles and the function of CLICs may involve enzymatic activity in the soluble form and anion channel activity in the integral membrane form^17^. For instance, currents flowing through intracellular Cl^−^ are crucial for the regulation of excitability in nerve and muscle^18, 19^. Further, bulk flow of chloride regulates cell volume and acidifies intracellular environment^20, 21^.

The possible correlation between CLICs and cardiovascular disease has been reported. In pulmonary arterial hypertension, CLIC4 gene deletion markedly attenuated the development of chronic hypoxia-induced pulmonary hypertension in mice, indicating that CLIC4 is a mediator of endothelial dysfunction in pulmonary hypertension^14^. In addition, CLIC5 is up-regulated in lungs from pulmonary hypertensive rats by proteomic studies^22^. In patients with nonischaemic dilated cardiomyopathy, reduced expression of CLIC3 is founded through microarray mRNA analysis^23^.

However, the role of CLICs and the correlation between CLICs and fibrotic changes of the atrium in the development of AF has not been reported. Moreover, whether CLICs play a role in the permanent AF associated with heart valve disease is unknown.

The purpose of the present study was to investigate the possible structure changes related to the mechanism of AF associated with heart valve disease at the tissue, mRNA, and protein levels by using transcriptomic and proteomic methods with attention on ion channels. Significantly differential mRNA and proteins related to ion channels and their correlation with fibrotic changes of the atrium were paid particular attention to.

## Results

### Patients’ characteristics

All patients had rheumatic heart valvular disease (RHD). In AF group, patients had sustained AF lasting for more than 6 months whereas in SR group (used as control) the patients were in sinus rhythm. The clinical characteristics of the patients were summarized in Supplementary Table 1. There were no significant differences between the AF and SR group regarding the demographical and baseline data. The example echocardiogtraphy images from the SR and AF group are shown in Supplementary Figure 1.

### Transcriptomic Study

**Figure 1:**
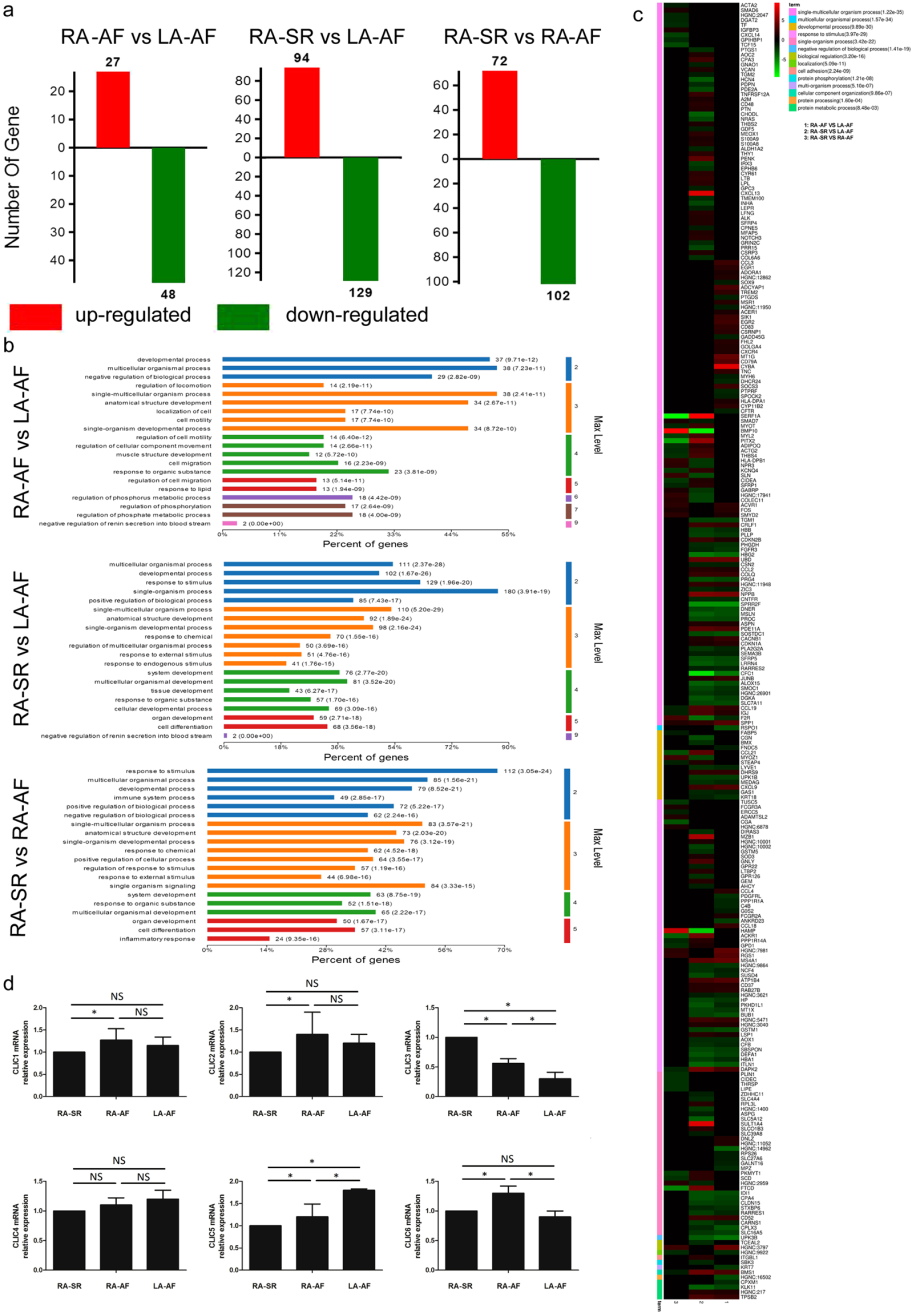
Analysis of transcriptome. (**a**) up-regulated and down-regulated genes in RA-AF vs. LA-AF, RA-SR vs. LA-AF and RA-SR vs. RA-AF. (**b**) The function of gene ontology consortium of the top 20 genes is shown among three groups. Horizontal ordinate represents the percent of genes enriched in biological function. Longitudinal ordinate represents biological function ranked according to *P* values. (**c**) 262 differentially expressed genes are identified, involving 14 functions (shown at the right upper corner). 1, RA-AF vs LA-AF, 2, RA-SR vs LA-AF, 3, RA-SR vs RA-AF. (**d**) mRNA expression of CLIC1, CLIC2, CLIC3, CLIC4, CLIC5 and CLIC6 among three groups. Compared to RA-SR, the expression of CLIC1, CLIC2, and CLIC5 was significantly up-regulated *(P* < 0.05 in the comparison between RA-AF and RA-SR for CLIC1, CLIC2, and CLIC5, between LA-AF and RA-SR or RA-AF for CLIC5). The expression of CLIC6 was upregulated in RA-AF (*P* < 0.05 compared to either RA-SR or LA-AF). In contrast, the expression of CLIC3 was significantly down-regulated in both RA-AF and LA-AF group (*P* < 0.05 compared to RA-SR). Further, CLIC3 was significantly lower in LA-AF than in RA-AF (*P* < 0.05). RA: right atrium; LA: left atrium; SR: sinus rhythm; AF: atrial fibrillation. *P < 0.05, NS: not significant.

We used RNA-Seq for the transcriptomic study in the right atrial tissue (RA-AF, n = 3) and left atrial tissue (LA-AF, n = 3) in AF patients and in the right atrial tissue in the patients with sinus rhythm (RA-SR, n = 2). We set the absolute value of log_2_Ratio ≥1 and probability ≥0.8 as filter condition. Compared to RA-SR, 94 genes were up-regulation and 129 genes were down-regulated in LA-AF. Similarly, 72 genes were up-regulation and 102 genes were down-regulated in RA-AF. In addition, 75 genes were differentially expressed between RA-AF and LA-AF (Fig. 1a). These genes are shown in the Supplementary Tables 2, 3, and 4.

Compared to RA-SR, 180 differential genes in LA-AF were related to single-organism process and 112 differential genes in RA-AF involved in the progress of response to stimulus in RA-AF (Fig. 1b).

There were 262 genes involved in well-defined functions, such as single-multicellular organism process, multicellular organismal process, developmental process, response to stimulus, single-organism process, negative regulation of biological process, biological regulation, localization, cell adhesion, protein phosphorylation, multi-organism process, cellular component organization, protein processing, and protein metabolic process (Fig. 1c).

The correlation between samples was shown in Supplementary Figure 2a. Genes are figured among individuals in Supplementary Figure 2b. In addition, top 20 statistics of pathway enrichment were analyzed. Among those, focal adhesion was significant, especially in RA-SR vs. LA-AF in Supplementary Figure 2c. The pathway is shown in the Supplementary Figure 3, redrawn from Kanehisal laboratories^24^. The raw data of transcriptome have been submitted to PubMed (BioProject ID: PRJNA392712).

### Differential expression of ion channels in the right and left atria in AF patients

Particular attention was paid to the RNA-Seq results regarding ion channels that are possibly related to AF such as the Ca^2+^, K^+^ and Na^+^ ion channels. However, no definitive findings regarding these ion channels could be discovered. The most striking findings from the RNA-Seq are the differentially expressed chloride channels, including CLICs and other chloride channels (see BioProject ID: PRJNA392712 for details). In combination with the results from the proteomic study (see below), CLICs were expressed differentially between the AF and SR patients and between the LA and RA tissue. Therefore, the subsequent studied were focused on CLICs.

### Validation of the differential expression of CLIC 1–6 mRNAs by qRT-PCR in the right and left atria in the new cohort of AF patients

After the expression of CLICs was confirmed by transcriptomics (Supplementary Table 5), we verified the expression of CLIC 1–6 mRNAs by qRT-PCR. Compared to RA-SR, CLIC1, CLIC2, and CLIC5 were significantly up-regulated (*P* < 0.05 in the comparison between RA-AF and RA-SR for CLIC1, CLIC2, and CLIC5, between LA-AF and RA-SR or RA-AF for CLIC5). The expression of CLIC6 was upregulated in RA-AF (*P* < 0.05 compared to either RA-SR or LA-AF). In contrast, the expression of CLIC3 was significantly down-regulated in both RA-AF and LA-AF group (*P* < 0.05 compared to RA-SR). Further, CLIC3 was significantly lower in LA-AF than in RA-AF (*P* < 0.05) (Fig. 1d).

### Proteomic study: identification of differentially expressed proteins in the right and left atria in AF patients

**Figure 2:**
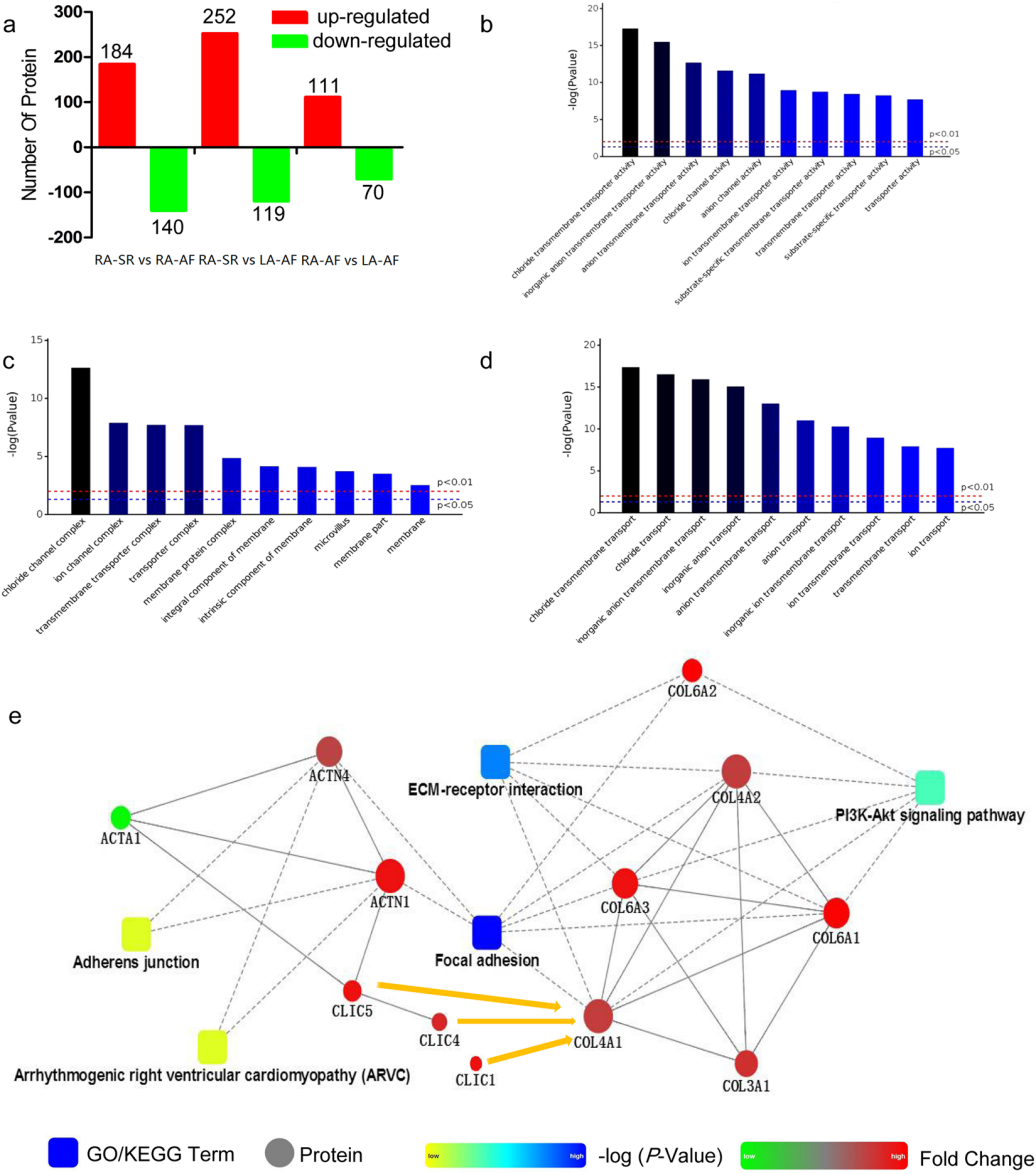
Analysis of proteomics. (**a**) up-regulated and down-regulated proteins in RA-AF vs. LA-AF, RA-SR vs. LA-AF, and RA-SR vs. RA-AF. (**b**) The function of CLIC1, 4 and 5 is shown regarding biological process, cell component, and molecular function. CLICs are involved in chloride transmembrane transporter activity, inorganic anion transmembrane transporter activity, anion transmembrane transporter activity, chloride channel activity, anion channel activity, ion transmembrane transporter activity, substrate-specific transmembrane transporter activity, transmembrane transporter activity, substrate-specific transporter activity and transporter activity. All these processes are significant (*P* < 0.01). (**c**) In cell component, CLICs are related to chloride channel complex, ion channel complex, transmembrane transporter complex, transporter complex, membrane protein complex, integral component of membrane, intrinsic component of membrane, microvillus, membrane part, and membrane. In contrast to RA-SR, cell component of CLICs in AF (RA-AF and LA-AF) groups are significant (*P* < 0.01). (**d**) CLICs are also participated in molecular function, including chloride transmembrane transport, chloride transport, inorganic anion transmembrane transport, inorganic anion transport, anion transmembrane transport, anion transport, inorganic ion transmembrane transport, ion transmembrane transport, transmembrane transport, ion transport. In contrast to RA-SR, the proteins were significantly different in the AF groups regarding biological processes, cell component and molecular function in AF group (P < 0.01). (**e**) Based on KEGG pathway database, the interaction of collagen, actinins (ACTN1 and ACTN4), and CLICs are demonstrated [48]. In this interaction, Type III, IV and VI collagen are participated in the extracellular matrix receptor interaction and PI3K-Akt signaling pathway. CLICs interacted with actinins, are involved in adherens junction and arrhythmogenic right ventricular cardiomyopathy. They are linked by the focal adhesion pathway, that is statistically significant for AF groups in both transcriptomics and proteomics. There is correlation between CLICs and type IV collagen (arrow). Note that all 6 types of CLICs were included in this analysis but only CLIC1, 4 and 5 remain in and other CLICs fall out from the interaction, suggesting the important role of CLIC1, 4 and 5.

The samples used in transcriptomics were also applied in the proteomic study by using iTraq technique. We set the cut-off 1.2 fold for up-regulation and 0.8-fold for down- regulation. Compared to RA-SR, 252 proteins were up-regulated in LA-AF and 140 proteins were down-regulated in RA-AF. Compared to RA-AF, there were 111 up-regulated proteins in LA-AF (Fig. 2a). These proteins were shown in the Supplementary Tables 6, 7, and 8. The raw data of proteome have been submitted to Proteome X change (Project accession: PXD006911).

Further, these differentially expressed proteins were categorized in biological process, cell component, and molecular function (Supplementary Figure 4). Among the pathways, similar to the transcriptomic study (see above), focal adhesion was also significant in the proteomic study (Supplementary Figure 3).

Although the RNAs of all 6 CLICs were differentially expressed in the AF and SR patients from the transcriptomic study (see above), in the proteomic study, only CLIC1, CLIC4, and CLIC5 proteins were differentially expressed between the AF and SR patients and between the LA and RA tissues. Therefore, in combination of the transcriptomics (Supplementary Table 9), qPCR validation, and the proteomics, the role of CLIC1, CLIC4, and CLIC5 proteins in AF was further analyzed.

### Function of CLICs

We analyzed the bioinformatics of CLICs regarding biological process, cell component, and molecular function. In biological process, CLICs are involved in chloride transmembrane transporter activity, inorganic anion transmembrane transporter activity, anion transmembrane transporter activity, and chloride channel activity etc. (Figure 2b). Regarding cell component, CLICs are related to chloride channel complex, ion channel complex, and transmembrane transporter complex etc. (Figure 2c). Regarding molecular function, CLICs are also involved in chloride transmembrane transport, chloride transport, and anion transport, etc. (Figure 2d). In contrast to RA-SR, the proteins were significantly different in the AF groups regarding biological processes, cell component and molecular function in AF group (*P* < 0.01).

### Possible correlation of CLICs and type IV collagen

The focal adhesion pathway is statistically significant in the AF group in both transcriptomic and proteomic studies. We further analyzed the possibility of protein-protein interaction between collagen and CLICs. As shown in Fig. 2, the collagens type III, IV, and VI are involved in extracellular matrix receptor interaction and PI3K-Akt signaling pathway. In adherens junction and arrhythmogenic right ventricular cardiomyopathy, CLICs interact with actinin (Fig. 2e). Interestingly, there is correlation between CLICs and the type IV collagen (see arrows in Fig. 2e).

### Distribution of the collagens type I, II, III, IV in atria

**Figure 3:**
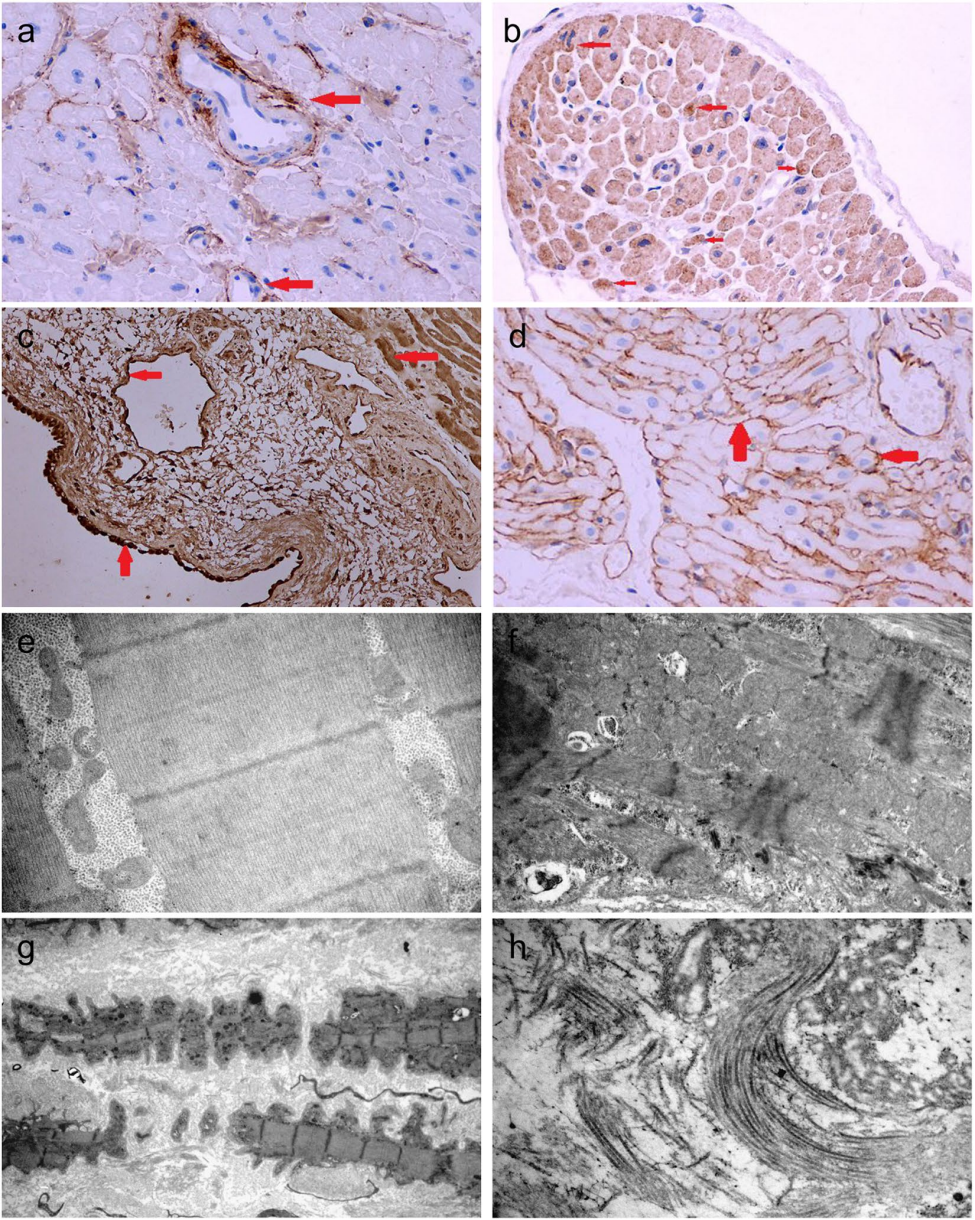
Immunohistochemistry staining with type I, II, III and IV collagen antibodies was used to identify their distribution in atrial tissue. Eight samples in each group were examined by electron microscopy and immunohistochemistry. The figure shows representative images from these experiments. (**a**) Type I collagen was found under capillary endothelial and vascular basement membrane. (**b**) Type II collagen was positive in cardiomyocytes and fibril cardiac muscle. (**c**) Type III collagen was colored in mesothelial cells, vascular endothelial cells, and myocyte cytoplasm. (**d**) Type IV collagen was located around cardiomyocytes, especially on vascular basement membrane and myocardial cell membrane. Red arrow indicated their location. Atrial tissue from SR and AF patients was observed by electron microscopy. (**e**) The structure of atrial myocyte was clear in SR patients, they connected mainly via intercalated discs. (**f**) In AF patients, mitochondrial swelling and vacuolization, loss of myofilament in cytoplasm of cardiomyocytes and widening intercalated discs. (**g**) In AF patients, atrial myocyte was necrosis; the connection of myocyte was disrupted. (**h**) At the intercellular space of atrial tissue in AF, collagen deposited, and immature fibrin was assembled and adhered to myocyte membrane.

Type I collagen staining was predominantly on the basement membrane, beneath the vascular endothelium and in the intercellular space (Fig. 3a). Myocardial collagen fiber was stained with type II collagen (Fig. 3b). Type III collagen was positive on capillary endothelial and vascular basement membrane (Fig. 3c). In contrast, type IV collagen was detected around myocyte, lipocyte, and vascular smooth muscle (Fig. 3d).

### Ultrastructure observation in AF

In RA-SR, atrial myocyte was connected with intercalated disc. Cardiomyocytes were tightly assembled with sarcomeres (Fig. 3e). In RA-AF and LA-AF, there were swelling mitochondria without cristae, mitochondrial vacuolization, and loss of myofilament in cardiomyocytes (Fig. 3f). In necrotic myocytes, collagen deposited in the intercellular space (Fig. 3g). Further, immature fibrin was assembled and adhered to the myocyte membrane (Fig. 3h).

### Validation of the differentially expressed CLIC1, 4, 5 and type IV collagen by Western blot in the right and left atria in AF patients

**Figure 4:**
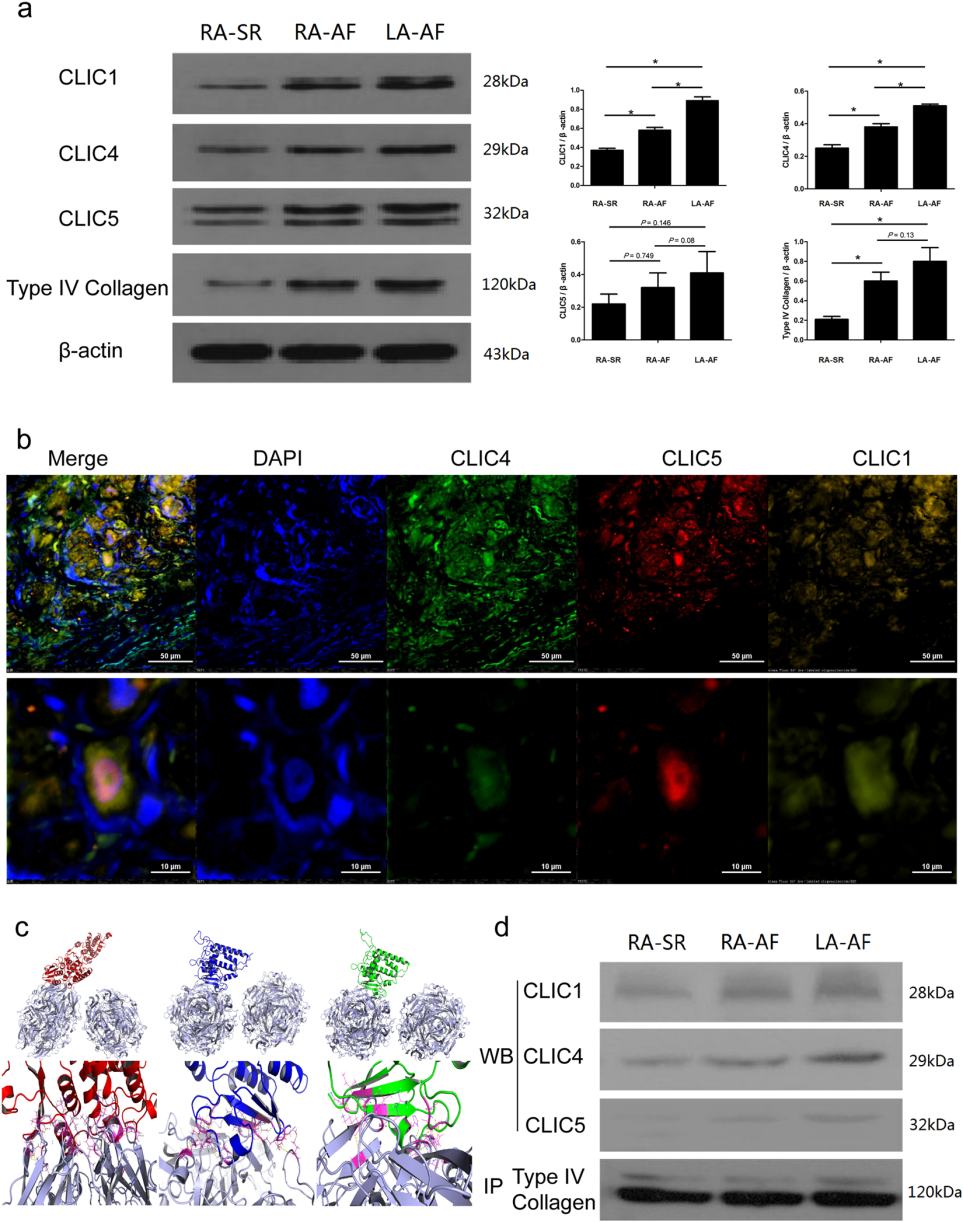
CLIC protein changes and interacts with type IV collagen in atrial tissue. (**a**) Western blot detected the expression of CLIC1, CLIC4, CLIC5 and type IV collagen protein in RA-SR (n = 26), RA-AF (n = 36) and LA-AF (n = 36). Beta-actin was used as the reference protein. The experiment was repeated for three times. For statistical analysis, the data were calculated for the value of mean ± S.D. One-way ANOVA was used for comparing the differences of data among groups. *P < 0.05. (**b**) IF-staining for CLIC1, CLIC4, and CLIC5 in the atrial tissue. DAPI for nuclear; Alexa Fluor 488 for detecting CLIC4, Alexa Fluor 594 for detecting CLIC5 and Alexa Fluor 633 for detecting CLIC1, far red light was adjusted to yellow one to distinguish with red light. (**c**) Computational protein structure prediction shows 3D structure of type IV collagen dimer (gray) and its binding site to CLIC1 (red), to CLIC4 (blue), and CLIC5 (green). (**d**) Interaction between the CLICs and type IV collagen was examined by co-immunoprecipitation. Compared to RA-SR, CLIC1, CLIC4 and CLIC5 combined with type IV collagen tightly in LA-AF and RA-AF.

The CLIC1, CLIC4, CLIC5 and type IV collagen were validated by Western blot. In RA-AF and LA-AF, the up-regulation of the expression of CLIC1 and CLIC4 was statistically different (*P* < 0.05). In contrast, CLIC5 was up-regulated in both RA-AF and LA-AF with no differences. Further, Type IV collagen was significantly up-regulated RA-AF, compared to RA-SR (*P* < 0.05) (Fig. 4a).

In addition, based on the Western blot findings, CLIC1, CLIC4, and CLIC5 in atrial tissue were analyzed by immunofluorescence staining. Figure 4b demonstrates that these channels are expressed in the atrial tissue widely, especially in nuclear periphery.

### Protein-protein docking between CLIC1, 4, 5 and type IV collagen

The computational program of protein-protein docking was employed to conduct molecular docking. The computation demonstrates that there was a tight binding between the protein of CLIC1, CLIC4, CLIC5, and type IV collagen. The maximum binding energy for type IV collagen with CLIC1, CLIC4, and CLIC5 was −124.58 kcal/mol, −128.809 kcal/mol, and −130.011 kcal/mol respectively. The binding sites for type IV collagen to CLIC1 were THR-75, LEU-30 and ASP-177 etc., to CLIC4 were ARG-169, MET-91 and ASPN-81 etc., and to CLIC5 were TYR-176, VAL-210 and GLU-87 etc. (Figure 4c).

In addition, the interaction between CLICs and type IV collagen was examined in RA-SR, RA-AF, and LA-AF by co-immunoprecipitation method. Figure 4d demonstrates the presence of interaction between CLICs and type IV collagen.

## Discussion

In this study, we have, for the first time, found that 1) alterations of CLIC1-6 by the RNA-Seq of transcriptomics and of CLIC1,4,5 by the iTRAQ of proteomics in the atrial tissue of rheumatic heart valve disease patients with AF in comparison to the patients with sinus rhythm. This indicates that chloride channels play an important role in the pathophysiology of AF; and 2) the up-regulation of type IV collagen may be a potential cause of abnormal activity of CLICs.

AF is one of the most common heart rhythm disorders, yet the regulatory molecular mechanisms underlying this syndrome are rather unclear. The underlying mechanisms of AF explored by microarray^25–27^ were related to cytoskeleton, immune/inflammatory response, fibrosis, ion channels, and etc. The electrical remodeling in sodium, potassium, and calcium ion channels contributes to the perpetuation of atrial fibrillation that refers to changes in ion channel gene transcription and protein expression, ion channel redistribution and development of fibrosis^28^. However, chloride channels have not been found to be related to the pathology of AF.

In the present study, the most astonishing finding is that the alteration of chloride channels is involved in the pathophysiology of AF in patients with heart valvular diseases. In the present study, by using RNA-seq technique, differential gene expression in patients with valvular heart disease and permanent AF was investigated and compared to that in patients with heart valvular disease and in sinus rhythm. Furthermore, at the basis of our previous proteomic studies in patients with valvular heart disease and congenital heart disease^29–33^, we used iTRAQ technique in the present study for proteomics in accordance with transcriptomics. The findings in the proteomics and transcriptomics were from the same sample, indicating the accordance in both gene and protein levels.

It should be aware of that there are possible differences between the left and right atria regarding gene expressions but no clear data are available. In the patients with sinus rhythm, we did not take LA tissue from the patients as control of the RA tissue due to ethical reasons. It cannot be completely ruled out that the differences seen between the left and right atria in the AF patients are possibly also related to the physiological differences. However, the comparison on the RA tissue between the patients on sinus rhythm and the patients with AF in this study provided solid data for the differences on gene expressions between these two groups of patients, suggesting the possible mechanism of AF related to CLIC channels.

The alterations of the chloride channels including CLIC1, CLIC4, and CLIC5 occur in both RNA-Seq and iTRAQ studies strongly support that the changes of chloride channels may play an important role in the pathophysiology of AF at least in heart valvular diseases. Interestingly, it was recently reported that only CLIC1, CLIC4, and CLIC5 were found in the rat heart^34^. The present study clearly demonstrates the existence of these CLICs in the human atria.

Functions of chloride channels on plasma membrane involves in ionic homeostasis, cell volume regulation, transepithelial transport and regulation of excitability, etc^35^. In cardiovascular diseases, previous studies showed that chloride channel was related to myocardial hypertrophy, ischemia, heart failure and hypertension^36^. Further, there is evidence that demonstrates significance of CLIC4 in apoptosis, cellular differentiation, inflammation, and endothelial tubulogenesis. In fact, CLIC4 expresses in vascular endothelial cells, cardiomyocytes, and lung alveolar septae, etc^37^. Furthermore, in the rodent heart, Ponnalagu and colleagues found that CLIC4 is enriched in the outer mitochondrial membrane, contrary to CLIC5 that are localized in the inner mitochondrial membrane^38^. It was reported that while being largely soluble proteins, CLICs can localise to cell membranes or lipid bilayers under specific conditions. Further, the localisation of CLIC proteins to cell membranes is often associated with processes that involve membrane remodelling mediated by the cortical actin cytoskeleton^17^. It has also been reported that CLIC proteins can spontaneously integrate into lipid bilayers. While direct integration of a soluble protein into a membrane is not a common property, there are large classes of proteins that possess this ability including bacterial pore forming toxins^39^, annexins and the Bcl-2 family of apoptotic proteins^40^.

By modulating mitochondrial function, chloride channels play an essential role in cardiac function and cardioprotection from ischemia-reperfusion injury. In addition to these findings, the present study demonstrates that the up-regulated CLIC1,4,5 are differentially expressed in the patients with AF. The results indicate that these chloride channels might affect atrial myocardial metabolism and homeostasis and therefore participate in the development of AF.

Collagen type I or type III deposition is one of the characteristics for fibrosis as well as tissue remodeling in AF patients, with numerous pathological validation studies confirming its validity^41–44^. Structural remodeling delays conduction velocity by disrupting intermyocyte coupling^45^. By using electron microscopy, we observed more immature collagen in the AF patients compared to SR patients. Thus, we speculate that collagen deposition is one of the causes of conduction delays in structural remodeling. Due to the fact that recent advancement in proteomic techniques has enabled the identification and quantification of specific collagen in AF, the results from the present study revealed that type IV collagen in LA-AF, in comparison to that in RA-SR, correlated better to the fibrosis in patients with rheumatic mitral valve disease and persistent AF. This was confirmed by both iTRAQ technique and the subsequent validation by using Western blot.

Although the structural and electrical remodeling are important mechanisms in AF, there are only few studies to elaborate their interaction. In Cx43 knockout mice, reduced expression of sodium channel accompanied increased expression of collagen resulting in slow and dispersed conduction for arrhythmia^46^. A recent study^47^ demonstrates that CLIC proteins have glutaredoxin-like glutathione-dependent oxidoreductase enzymatic activity that gives some clue for possible effect of CLIC proteins distinct from the channel. Although these studies did not discuss the interaction between ion channel function and structure remodeling in AF, they may suggest that ion channel abnormalities and atrial remodeling should have intrinsic links. Here, we have found that there is a stronger interaction between chloride channels and type IV collagen in AF than in SR, suggesting that the fibrosis in AF not only leads to the structural remodeling, but also affects electrical activity. Thus, we believe that the association between type IV collagen and CLICs is an important mechanism in the development of AF.

The present study provides basic concept on the role of chloride channels of atrial myocytes in the development of AF in the rheumatic heart valve disease. The findings from the present study promote further functional studies and transgenic mice studies as to the role of chloride channels in the mechanism of the development of AF. Further, although the results are from the patients with AF in rheumatic heart valve disease, these findings may have implications more widely with regard to the mechanism in other types of AF.

In conclusion, our study suggests that the chloride channels as a potential electrical remodeling factor are closely linked to the development of AF in rheumatic heart valve disease. Further, it is possible that the up-regulation of type IV collagen may also be a characteristic feature of AF in rheumatic mitral valve disease. The findings from the present study in atrial remodeling and electrical changes may provide a new insight for the mechanism of development of AF.

## Methods

### Diagnosis of Heart Valve Disease

Sixty-two consecutive patients with rheumatic heart valve disease were enrolled in the present study.

### Diagnosis of AF

Human atrial tissues were taken from the patients with rheumatic mitral valve disease in either sinus rhythm or persistent AF. The diagnosis of AF was mainly made by persistent AF from EKG by multiple recordings. SR patients were repeatedly examined to ensure that they had never experienced AF, by direct questioning about symptoms suggestive of AF and by multiple 12-lead electrocardiography during their entire preoperative review period.

### Echocardiography

All patients had multiple transthoracic echocardiography (TTE) examinations prior to surgery as the usual practice. All examinations were performed by experienced echocardiographers specialized in cardiac echocardiography. The size and shape of heart valves and chambers were measured and recorded. In all cases, the severity of the mitral valve stenosis or regurgitation, the quality of the valve leaflets, particularly the size of the left atrium, as well as the details of other valves and cardiac chambers were recorded. During the operation for valve replacement, transesophageal echocardiography (TEE) was performed routinely prior to the opening of the chest to determine the accuracy of the preoperative TTE. TEE was performed again immediately after the valve replacement when cardiopulmonary bypass was ceased to check the status of the artificial valve and the cardiac function.

### Human Tissue Samples

Patients were divided into AF group (n = 36, AF > 6 months before surgery) and SR group (n = 26, without history of AF). In AF patients, right atrial (RA) and Left atrial (LA) tissues were obtained during the valve replacement surgery. The exact area that the tissue was taken from was: the LA tissue was mainly taken from the LA near the pulmonary veins by the senior author who normally does mitral valve replacement through a LA incision and his colleagues; the RA tissue was also taken from either the RA incision or the bottom of the RA appendage at TEDA international cardiovascular hospital. The reason not to collect the LA tissue in the patients with sinus rhythm is because for those patients, there was no procedure performed in the LA. All work with human samples conforms to the Declaration of Helsinki and procedures were approved by the Institutional Review Board of TEDA International Cardiovascular Hospital. The informed consent was given prior to the inclusion of the patient before surgery. Patients with familial paroxysmal atrial fibrillation, hyperthyreosis, sick sinus syndrome, pulmonary heart disease, cardiomyopathy, renal disease, and secondary thoracotomy were excluded from the study.

### Surgical Procedure

The operation was performed under general anesthesia and cardiopulmonary bypass under mild to moderate hypothermia. Blood cardioplegia was used to arrest the heart. The aorta was cross-clamped and the operation for mitral valve replacement was through either left atrium or atrial septum. Mechanical valve prostheses were used for patients under 60 year-old. For those older than 60, bioprostheses or mechanical valve prostheses were used according to the patient’s choice and other conditions. In the patients associated with AF, the LA appendage was usually resected with/without simultaneous radio frequency ablation (modified Maze Procedure). The patient was in ICU usually for 2 days and routine treatment for post-valve replacement was followed.

### Transcriptomic Studies

In this part of study, mRNA isolation, cDNA library construction, and sequencing were performed by the Beijing Genomics Institute (BGI) (Shenzhen, China). Briefly, total RNA was extracted from each tissue using TRIzol reagent (Invitrogen, Burlington, ON, Canada) and digested with DNase I (Takara, Dalian, China) according to the manufacturer’s protocol. Next, Oligo (dT) magnetic beads were used to isolate mRNA from the total RNA. By mixing with fragmentation buffer, the mRNA was then broken into short fragments. The cDNA was synthesized using the mRNA fragments as templates. The short fragments were purified and resolved with EB buffer for end repair and single nucleotide A (adenine) addition, and then connected with adapters. Suitable fragments were selected for PCR amplification as templates. During the quality control steps, an Agilent 2100 Bioanalyzer (Agilent Technologies, Redwood City, CA, USA) and ABI StepOnePlus Real-Time PCR System (Life Technologies, Grand Island, NY, USA) were used for quantification and qualification of the sample library. Each cDNA library was sequenced in a single lane of the Illumina HiSeqTM 2000 system using paired end protocols according to the manufacturer’s instructions at BGI.

### Proteomic Studies

The samples eluted by Lysis buffer were reduced with 10 mmol/L DTT at 56 °C for 60 min and then alkylated. The protein mixtures were precipitated by precooled acetone at −20 °C overnight, and then centrifuged at 4 °C, 30,000 g for 30 min. The pellet was dissolved in 0.5 mmol/L TEAB (Applied Biosystems, Milan, Italy) and sonicated in ice. After a centrifuging at 4 °C, 30,000 g, the supernatant was used for liquid digestion with Trypsin Gold (Promega, Madison, WI, USA). The peptides were dried and reconstituted in 0.5 mmol/L TEAB and mixed with 70ul of isopropanol. Samples were labeled with iTRAQ reagent (Applied Biosystems, Milan, Italy). The peptides were dried and dissolved in 4 ml of buffer A (25 mmol/L NaH_2_PO_4_ in 25% CAN, pH 2.7). The sample was fractionated using cation-exchange chromatography (SCX) on a LC-20AB HPLC Pump system (Shimadzu, Kyoto, Japan). The peptides were eluted at a flow rate of 1 mL/min with a gradient of buffer A for 10 min, 5–60% buffer B (25 mmol/L NaH_2_PO_4_ and 1 mol/L KCl in 25% ACN, pH 2.7) for 27 min, 60–100% buffer B for 1 min. The fractions were desalted and dried. Then, buffer A (5% ACN, 0.1% FA) was added to each dried fraction tube, and 10 ul supernatant of the re-dissolved solution was loaded on a LC-20AD nanoHPLC (Shimadzu, Kyoto, Japan) and separated over a 35 min gradient from 2 to 35% in 0.1% FA combined with 95% ACN. Data acquisition were performed with a TripleTOF 5600 System (AB SCIEX, Concord, ON). Proteins identification was performed by using Mascot search engine (Matrix Science, London, UK; version 2.3.02). The quantitative protein ratios were weighted and normalized by the median ratio in Mascot. The fold changes of >1.2 (or <0.8) and *P* value less than 0.05 were considered as significant.

### Quantitative RT-PCR

To analyze the mRNA levels of CLIC1, CLIC2, CLIC3, CLIC4, CLIC5, and CLIC6 in each group, total mRNA was isolated from atrial tissue using the PureLink™ RNA Mini Kit (Life Technologies). Subsequently, the mRNA was reverse-transcribed using TaqMan Reverse Transcription Reagents (Applied Biosystems). Quantitative real-time reverse transcription– polymerase chain reactions (RT-qPCR) were performed using LightCycler^®^96 and the primers listed in Supplementary Table 9. The fold changes of expression of each target mRNA relative to GAPDH under experimental conditions compared to the control conditions were calculated based on the threshold cycle (C_T_) as follows: r = 2^−Δ(ΔCT)^, where ΔC_T_ = C_T_(target) − C_T_(GAPDH) and Δ(ΔC_T_) = ΔC_T_(experimental) − ΔC_T_(control).

### Western Blot

Protein samples (20 μg of total protein) were mixed with SDS-PAGE gel loading buffer supplemented with 5% of β-mercaptoethanol and incubated at 100 °C for 5 minutes before being loaded on 6% polyacrylamide gels. After electrophoresis, proteins were transferred to nitrocellulose membranes. Duplicate gels were run and blotted. Membranes were probed with antibodies raised against the CLIC1 (1:200, Santa Cruz, sc-51048), CLIC4 (1:400, Santa Cruz, sc-135739), CLIC5 (1:200, Santa Cruz, sc-133468), type IV collagen (1:1000, Abcam, ab6586), and β-actin (1:200, Santa Cruz, sc-130065). After extensive washes, the membranes were incubated with HRP-conjugated secondary antibody and detected using the Immun-Star HRP Substrate Kit (Bio-Rad). Quantification of the signals on the membranes was carried out by using Image J program.

### Immunofluorescent Staining

Serial cutting sections were used for immunofluorescent staining. Formalin-fixed, paraffin-embedded tissue sections were serially rehydrated in 100, 95, and 70% ethanol after deparaffinization with xylene. The antigen retrieval procedures were then performed. The slides were microwaved in 10 mmol/L citrate buffer (pH = 6.0) for 3 min. After 30 min of cooling, the slides were washed in PBS 3 times for 5 min each. The tissue sections were incubated in a blocking solution containing 10% goat serum and 1% bovine serum albumin in PBS to reduce nonspecific binding. The sections were incubated with CLIC1 (1:200; Santa Cruz), CLIC4(1:400; Santa Cruz), and CLIC5 (1:400; Santa Cruz) overnight at 4 °C. The sections were then incubated for 90 min at room temperature with an Alexa Fluor 488 donkey anti-mouse immunoglobulin G, an Alexa Fluor 594 donkey anti-rabbit immunoglobulin G or an Alexa Fluor 633 donkey anti-goat immunoglobulin G. The nuclei were counterstained with DAPI (2.5 μg/ml in PBS; Molecular Probes). The slides were visualized using a Leica TCS SP5 confocal microscope.

### Electron Microscopy

Fresh specimens were cut into pieces (2 × 2 mm) and fixed in 2% glutaraldehyde (pH = 7. 4) for 6–8 h at 4 °C. They were washed and post fixed in 1% OsO_4_for 1 h, at 4 °C. The tissue was dehydrated through ascending grades of ethanol and embedded in araldite CY212. Semi thin sections (1 μm) were cut and stained with toluidine blue. Ultra-thin sections (60–70 nm) were cut and stained with uranyl acetate and alkaline lead citrate.

### Immunohistochemistry

As previously reported^48^, the sections were washed in PBS and incubated separately overnight with the primary antibodies for the type I, II, III and IV collagen. After incubation, the sections were washed in PBS and incubated in the vectastain avidin-biotin complex (ABC) kit for 1.5 h. The slides were then washed and mounted for light microscopy.

### Protein Structure Prediction by Computational Program of Protein-Protein Docking

Molecular docking using the atomic coordinates of CLIC1, CLIC4 and Type IV collagen were downloaded from a protein databank (PDB). The crystal structure of CLIC5 was not resolved. We downloaded the simulated structure from Protein Model Portal. The computational program of protein-protein docking was employed to conduct molecular docking. The protein structures were prepared by adding hydrogen, treating disulfides, assigning bond order, assigning protonation states, and performing energy minimization to relax the structures. The minimization was terminated when the root-mean-square deviation (RMSD) reached a maximum cutoff of 0.3 Å. In the docking process, docking parameters were set to the default of software.

### Co-immunoprecipitation

Tissue samples were lysed with lysis buffer and PMSF. Type IV Collagen antibody (1:100, Abcam) was then added in the lysate and shaked at 4 °C overnight. Protein A + G Agarose (Beyotime Biotechnology, China) was added for 4 h and then centrifuged at 500 g, 5 min for 5 times. Next, equivalent protein amounts were denatured in an SDS sample buffer and were ready for the Western blot analysis.

### Statistical Analysis

All data were analyzed using SPSS 17.0. The data are reported as the mean ± S.D. Significant differences with respect to patients’ characteristics were analyzed using independent sample t-test, Chi-square or Fisher’s exact test. Data among groups were compared by One-way ANOVA test. *P* less than 0.05 was considered significant. The bioinformatic analysis was performed by BGI and Shanghai Bo-Yuan Biotechnology CO., Ltd.

## Electronic supplementary material

supplementary materials
