# Supplementary material for: Chloride Channels are Involved in the Development of Atrial Fibrillation – A Transcriptomic and proteomic Study
Source: Sci Rep. 2017 Aug 31;7:10215. doi: 10.1038/s41598-017-10590-w (PMC5579191; doi:10.1038/s41598-017-10590-w)
Supplement: Supplementary file 1 — supplementary materials [file 41598_2017_10590_MOESM1_ESM.pdf]

Chloride Channels Are Involved in the Development of Atrial Fibrillation – A Transcriptomic and proteomic Study

Yi-Yao Jiang, Hai-Tao Hou, Qin Yang, Xiao-Cheng Liu, and *Guo-Wei He

Supplementary Table 1. Patients Characteristics

	AF	SR	*P* value
Patients, n	36	26	
Gender (M:F)	5:4	15:11	
Age (years)	59.53±8.17	51.38±13.30	NS
BMI (kg/m^2)	23.98±4.21	23.5±2.57	NS
LA (mm)	53.93±8.02	49.63±6.94	NS
LV (mm)	55.475±7.16	54.25±8.53	NS
RA (mm)	40.40±4.32	36.63±3.07	NS
RV (mm)	36.40±3.18	33.25±3.45	NS
LVEF%	58.42±4.46	62.25±4.30	NS
Hypertension, n	4	2	NS
Hyperlipidaemia, n	3	1	NS
Diabetes, n	2	1	NS
ACE inhibitors, n	2	1	NS
AT1-blockers, n	0	1	NS
Nitrates, n	5	2	NS
Lipid-lowering drugs, n	3	1	NS
Ca^{2+}-antagonists, n	2	1	NS

BMI: body mass index. LA: left atria. LV: left ventricular. RA: right atria. RV: right ventricular. NS: No Significance

Chloride Channels Are Involved in the Development of Atrial Fibrillation – A Transcriptomic and proteomic Study

Yi-Yao Jiang, Hai-Tao Hou, Qin Yang, Xiao-Cheng Liu, and *Guo-Wei He

Supplementary Table 2. The differential expression between RA-SR and LA-AF

RA-SR vs LA-AF			
Up-regulation		Down-regulation	
Gene ID	Symbol	Gene ID	Symbol
5308	PITX2	55997	CFC1
7503	XIST	3880	KRT19
64499	TPSB2	1271	CNTFR
3955	LFNG	5354	PLP1
56901	NDUFA4L2	51200	CPA4
1311	COMP	2775	GNAO1
948	CD36	93099	DMKN
80303	EFHD1	100216479	LOC100216479
2819	GPD1	84952	CGNL1
51330	TNFRSF12A	374569	ASPG
3512	IGJ	7348	UPK1B
951	CD37	92737	DNER
6347	CCL2	57211	GPR126
6363	CCL19	9628	RGS6
5764	PTN	9077	DIRAS3
9383	TSIX	7052	TGM2
72	ACTG2	80761	UPK3B
1043	CD52	100130827	SGK110
3936	LCP1	5320	PLA2G2A
8048	CSRP3	55273	TMEM100
4046	LSP1	57699	CPNE5
10578	GNLY	3623	INHA
2	A2M	629	CFB
100423062	IGLL5	27302	BMP10
4050	LTB	10021	HCN4
5179	PENK	2051	EPHB6
10563	CXCL13	5918	RARRES1
1359	CPA3	64093	SMOC1
6818	SULT1A3	1949	EFNB3
6422	SFRP1	140597	TCEAL2
962	CD48	131873	COL6A6
9510	ADAMTS1	3048	HBG2
3111	HLA-DOA	2949	GSTM5
3485	IGFBP2	93035	PKHD1L1

6123	RPL3L	4883	NPR3
9244	CRLF1	9254	CACNA2D2
7060	THBS4	8991	SELENBP1
94274	PPP1R14A	79191	IRX3
51237	MZB1	6705	SPRR2F
4222	MEOX1	11012	KLK11
931	MS4A1	25928	SOSTDC1
6366	CCL21	54839	LRRC49
7058	THBS2	64073	C19orf33
1149	CIDEA	29091	STXBP6
4023	LPL	244	ANXA8L2
347	APOD	7051	TGM1
100874095	ADIPOQ-AS1	79748	LMAN1L
6279	S100A8	126661	CCDC163P
388524	RPSAP58	157869	SBSPON
2669	GEM	26227	PHGDH
728978	UNC5B-AS1	660	BMX
8076	MFAP5	794	CALB2
23439	ATP1B4	55061	SUSD4
22822	PHLDA1	2619	GAS1
2532	DARC	58529	MYOZ1
8292	COLQ	10630	PDPN
1026	CDKN1A	745	MYRF
7138	TNNT1	2845	GPR22
6696	SPP1	10158	PDZK1IP1
618	BCYRN1	7869	SEMA3B
3726	JUNB	6588	SLN
9358	ITGBL1	419	ART3
6649	SOD3	653145	ANXA8
7177	TPSAB1	92293	TMEM132C
1277	COL1A1	5919	RARRES2
79689	STEAP4	3875	KRT18
3164	NR4A1	3043	HBB
2003	ELK2AP	4092	SMAD7
100008587	RNA5-8S5	646	BNC1
10537	UBD	55600	ITLN1
563	AZGP1	2905	GRIN2C
6319	SCD	389125	MUSTN1
9499	MYOT	3040	HBA2
4854	NOTCH3	133	ADM
79742	CXorf36	6425	SFRP5
9370	ADIPOQ	159963	SLC5A12
780851	SNORD3A	84935	MEDAG
2167	FABP4	5742	PTGS1

4633	MYL2	2944	GSTM1
1462	VCAN	78989	COLEC11
1827	RCAN1	144809	FAM216B
4283	CXCL9	3953	LEPR
54829	ASPN	114569	MAL2
10170	DHRS9	3240	HP
6280	S100A9	316	AOX1
6424	SFRP4	10216	PRG4
8490	RGS5	342527	SMTNL2
7070	THY1	23657	SLC7A11
8293	SERF1A	780	DDR1
51208	CLDN18	57571	CARNS1
1114	CHGB	5947	RBP1
4053	LTBP2	379	ARL4D
4879	NPPB	90187	EMILIN3
3491	CYR61	57530	CGN
		4501	MT1X
		2719	GPC3
		151354	FAM84A
		57451	TENM2
		24146	CLDN15
		10232	MSLN
		79844	ZDHHC11
		8685	MARCO
		140578	CHODL
		6860	SYT4
		3039	HBA1
		728113	ANXA8L1
		590	BCHE
		164312	LRRN4
		50863	NTM
		8671	SLC4A4
		246	ALOX15
		142683	ITLN2
		1776	DNASE1L3
		27184	DISC2
		100132708	CYP4F30P
		283310	OTOGL
		3250	HPR
		10894	LYVE1
		8854	ALDH1A2
		57817	HAMP
		3426	CFI
		1297	COL9A1

	64116	SLC39A8
	56961	SHD
	594855	CPLX3
	51090	PLLP
	6590	SLPI
	222171	PRR15
	2823	GPM6A

Chloride Channels Are Involved in the Development of Atrial Fibrillation – A Transcriptomic and proteomic Study

Yi-Yao Jiang, Hai-Tao Hou, Qin Yang, Xiao-Cheng Liu, and *Guo-Wei He

Supplementary Table 2. The differential expression between RA-SR and RA-AF

RA-SR vs RA-AF			
Up-regulation		Down-regulation	
Gene ID	Symbol	Gene ID	Symbol
245806	VGLL2	55997	CFC1
7503	XIST	3880	KRT19
18	ABAT	1271	CNTFR
1959	EGR2	51200	CPA4
7852	CXCR4	5502	PPP1R1A
1311	COMP	100216479	LOC100216479
3512	IGJ	252995	FNDC5
951	CD37	5730	PTGDS
6347	CCL2	7348	UPK1B
2354	FOSB	92737	DNER
6363	CCL19	80761	UPK3B
9560	CCL4L1	5320	PLA2G2A
9383	TSIX	629	CFB
5996	RGS1	5918	RARRES1
3371	TNC	50486	G0S2
1043	CD52	23213	SULF1
3936	LCP1	64093	SMOC1
2274	FHL2	1949	EFNB3
4046	LSP1	10912	GADD45G
973	CD79A	3048	HBG2
9021	SOCS3	93035	PKHD1L1
100423062	IGLL5	8991	SELENBP1
54209	TREM2	4359	MPZ
6348	CCL3	6705	SPRR2F
1958	EGR1	11012	KLK11
4929	NR4A2	25928	SOSTDC1
9510	ADAMTS1	64073	C19orf33
6533	SLC6A6	29091	STXBP6
3485	IGFBP2	284654	RSPO1
9244	CRLF1	244	ANXA8L2
56950	SMYD2	7051	TGM1
931	MS4A1	79748	LMAN1L
6515	SLC2A3	126661	CCDC163P
6362	CCL18	157869	SBSPON

388524	RPSAP58	26227	PHGDH
25878	MXRA5	92346	C1orf105
728978	UNC5B-AS1	4071	TM4SF1
4481	MSR1	3855	KRT7
23439	ATP1B4	794	CALB2
22822	PHLDA1	55061	SUSD4
2212	FCGR2A	157310	PEBP4
6351	CCL4	9806	SPOCK2
150094	SIK1	2619	GAS1
6349	CCL3L1	745	MYRF
8292	COLQ	57452	GALNT16
1026	CDKN1A	10158	PDZK1IP1
7138	TNNT1	57586	SYT13
6696	SPP1	7869	SEMA3B
618	BCYRN1	4500	MT1L
3726	JUNB	653145	ANXA8
7177	TPSAB1	28965	SLC27A6
81606	LBH	5919	RARRES2
9308	CD83	3875	KRT18
3164	NR4A1	3043	HBB
2003	ELK2AP	646	BNC1
100008587	RNA5-8S5	55600	ITLN1
10537	UBD	5157	PDGFRL
335	APOA1	343263	MYBPHL
780851	SNORD3A	389125	MUSTN1
2803	GOLGA4	3040	HBA2
1827	RCAN1	6425	SFRP5
4283	CXCL9	84935	MEDAG
4495	MT1G	2944	GSTM1
54829	ASPN	5950	RBP4
10170	DHRS9	4624	MYH6
728489	DNLZ	114569	MAL2
7538	ZFP36	7140	TNNT3
3113	HLA-DPA1	3240	HP
64651	CSRNP1	316	AOX1
1114	CHGB	10216	PRG4
2353	FOS	342527	SMTNL2
4879	NPPB	23657	SLC7A11
		57571	CARNS1
		5947	RBP1
		5792	PTPRF
		379	ARL4D
		718	C3
		4501	MT1X

56265	CPXM1
1718	DHCR24
24146	CLDN15
10232	MSLN
8685	MARCO
162494	RHBDL3
6860	SYT4
3039	HBA1
728113	ANXA8L1
590	BCHE
164312	LRRN4
246	ALOX15
142683	ITLN2
100293534	C4B_2
100132708	CYP4F30P
283310	OTOGL
10894	LYVE1
3426	CFI
56961	SHD
594855	CPLX3
51090	PLLP
6590	SLPI
200539	ANKRD23
6231	RPS26

Chloride Channels Are Involved in the Development of Atrial Fibrillation – A Transcriptomic and proteomic Study

Yi-Yao Jiang, Hai-Tao Hou, Qin Yang, Xiao-Cheng Liu, and *Guo-Wei He

Supplementary Table 4. The differential expression between RA-AF and LA-AF

RA-AF vs LA-AF			
Up-regulation		Down-regulation	
Gene ID	Symbol	Gene ID	Symbol
84649	DGAT2	2354	FOSB
5308	PITX2	27302	BMP10
2819	GPD1	5996	RGS1
3512	IGJ	167681	PRSS35
6363	CCL19	2214	FCGR3A
286753	TUSC5	4883	NPR3
7122	CLDN5	4929	NR4A2
72	ACTG2	56950	SMYD2
100423062	IGLL5	58529	MYOZ1
6422	SFRP1	6588	SLN
7060	THBS4	419	ART3
94274	PPP1R14A	4092	SMAD7
6366	CCL21	6696	SPP1
63924	CIDEC	9719	ADAMTSL2
1149	CIDEA	133	ADM
347	APOD	5596	MAPK4
100874095	ADIPOQ-AS1	78989	COLEC11
6876	TAGLN	3486	IGFBP3
84417	C2orf40	335	APOA1
2532	DARC	57451	TENM2
338328	GPIHBP1	8685	MARCO
7018	TF	4091	SMAD6
343263	MYBPHL	50863	NTM
9547	CXCL14	1776	DNASE1L3
1466	CSRP2	27184	DISC2
4504	MT3	57817	HAMP
5346	PLIN1	2353	FOS
5950	RBP4		
79689	STEAP4		
2003	ELK2AP		
563	AZGP1		
6319	SCD		
6939	TCF15		
9499	MYOT		

79762	C1orf115
79742	CXorf36
9370	ADIPOQ
2167	FABP4
4633	MYL2
3991	LIPE
7069	THRSP
8293	SERF1A
2171	FABP5
153579	BTNL9
51208	CLDN18
1114	CHGB
59	ACTA2
338872	C1QTNF9

Chloride Channels Are Involved in the Development of Atrial Fibrillation – A Transcriptomic and proteomic Study

Yi-Yao Jiang, Hai-Tao Hou, Qin Yang, Xiao-Cheng Liu, and *Guo-Wei He

Supplementary Table 5. The differential expression of CLICs in transcriptome

Gene ID	Means SR	Means AFR	log_2Ratio (RA-AF/RA-SR)	Symbol
1192	82	87.2	0.0887	CLIC1
1193	8.835	9.377	0.0858	CLIC2
9022	5.595	2.667	-1.0691	CLIC3
25932	91.945	107.287	0.2226	CLIC4
53405	44.31	57.297	0.3708	CLIC5
54102	7.78	9.277	0.2538	CLIC6

Gene ID	Means SR	Means AFL	log_2Ratio (LA-AF/RA-SR)	Symbol
1192	82	93.24	0.185	CLIC1
1193	8.835	9.516	0.107	CLIC2
9022	5.595	2.883	-0.956	CLIC3
25932	91.945	106.92	0.217	CLIC4
53405	44.31	44.297	-0.0004	CLIC5
54102	7.78	5.71	-0.446	CLIC6

Gene ID	Means AFL	Means AFR	log_2Ratio (RA-AF/LA-AF)	Symbol
1192	93.24	87.2	-0.097	CLIC1
1193	9.517	9.377	-0.021	CLIC2
9022	2.88	2.667	-0.113	CLIC3
25932	106.92	107.287	0.005	CLIC4
53405	44.297	57.297	0.371	CLIC5
54102	5.71	9.277	0.700	CLIC6

Chloride Channels Are Involved in the Development of Atrial Fibrillation – A Transcriptomic and proteomic Study

Yi-Yao Jiang, Hai-Tao Hou, Qin Yang, Xiao-Cheng Liu, and *Guo-Wei He

Supplementary Table 6. The differential expression between RA-SR and LA-AF

RA-SR vs LA-AF	
Up-regulation	Down-regulation
tr|M0QXF9|M0QXF9_HUMAN	tr|A0A024R9U8|A0A024R9U8_HUMAN
sp|P31146|COR1A_HUMAN	sp|Q9UBG0|MRC2_HUMAN
tr|A8K1D2|A8K1D2_HUMAN	sp|P08727|K1C19_HUMAN
sp|Q07960|RHG01_HUMAN	tr|B7Z5J1|B7Z5J1_HUMAN
sp|Q15942|ZYX_HUMAN	sp|P16157|ANK1_HUMAN
sp|Q9NZU5|LMCD1_HUMAN	tr|B4DNG0|B4DNG0_HUMAN
sp|P05109|S10A8_HUMAN	tr|D3GKD8|D3GKD8_HUMAN
tr|H0YCP8|H0YCP8_HUMAN	sp|P23142|FBLN1_HUMAN
tr|B4DVZ0|B4DVZ0_HUMAN	tr|Q8N7G1|Q8N7G1_HUMAN
sp|P26885|FKBP2_HUMAN	tr|B7Z8A2|B7Z8A2_HUMAN
tr|B2R941|B2R941_HUMAN	tr|A5YM48|A5YM48_HUMAN
sp|O43707|ACTN4_HUMAN	tr|J3KQY1|J3KQY1_HUMAN
tr|J3KQ99|J3KQ99_HUMAN	sp|O95210|STBD1_HUMAN
sp|P12110|CO6A2_HUMAN	tr|B7Z4A1|B7Z4A1_HUMAN
sp|Q8WW22|DNJA4_HUMAN	tr|B4E1B2|B4E1B2_HUMAN
sp|Q9UMX5|NENF_HUMAN	sp|Q5BKX8|MURC_HUMAN
sp|Q03135|CAV1_HUMAN	tr|B4DL07|B4DL07_HUMAN
tr|B3KM80|B3KM80_HUMAN	tr|H0YL90|H0YL90_HUMAN
tr|B3KRN4|B3KRN4_HUMAN	tr|L8E853|L8E853_HUMAN
tr|H0Y9H2|H0Y9H2_HUMAN	tr|A0A024R2W4|A0A024R2W4_HUMAN
sp|Q06323|PSME1_HUMAN	sp|P43652|AFAM_HUMAN
tr|B3KM68|B3KM68_HUMAN	tr|A2RRE0|A2RRE0_HUMAN
sp|P23381|SYWC_HUMAN	tr|H6VRF8|H6VRF8_HUMAN
sp|P04004|VTNC_HUMAN	sp|Q6ZP80|TM182_HUMAN
tr|B7Z6G4|B7Z6G4_HUMAN	tr|B4DMD3|B4DMD3_HUMAN
tr|Q59HA3|Q59HA3_HUMAN	sp|Q9Y584|TIM22_HUMAN
tr|H0YGX7|H0YGX7_HUMAN	tr|B2R6F7|B2R6F7_HUMAN
tr|G3V3X5|G3V3X5_HUMAN	tr|B4DI75|B4DI75_HUMAN
tr|Q6DEN2|Q6DEN2_HUMAN	tr|B0AZQ4|B0AZQ4_HUMAN
sp|P43490|NAMPT_HUMAN	sp|Q9NP98|MYOZ1_HUMAN
sp|Q9BX66|SRBS1_HUMAN	sp|P05546|HEP2_HUMAN
tr|B3KVC9|B3KVC9_HUMAN	sp|P05154|IPSP_HUMAN
tr|M0QYT0|M0QYT0_HUMAN	sp|P30043|BLVRB_HUMAN
sp|P15121|ALDR_HUMAN	sp|Q99959|PKP2_HUMAN
tr|Q6FIE5|Q6FIE5_HUMAN	sp|Q9UK22|FBX2_HUMAN

sp|Q9NZA1|CLIC5_HUMAN
sp|P13929|ENOB_HUMAN
tr|A0A087WXM6|A0A087WXM6_HUMAN
tr|A0A024R493|A0A024R493_HUMAN
tr|F8WCF6|F8WCF6_HUMAN
sp|O00151|PDLI1_HUMAN
sp|Q02338|BDH_HUMAN
sp|P07437|TBB5_HUMAN
tr|Q9UL89|Q9UL89_HUMAN
tr|H0Y614|H0Y614_HUMAN
tr|A0A024R091|A0A024R091_HUMAN
tr|Q5RKT7|Q5RKT7_HUMAN
sp|P61586|RHOA_HUMAN
sp|O95433|AHSA1_HUMAN
tr|E7EMG9|E7EMG9_HUMAN
tr|W6A4U0|W6A4U0_HUMAN
sp|P61604|CH10_HUMAN
tr|B4DND4|B4DND4_HUMAN
tr|B4DUJ6|B4DUJ6_HUMAN
tr|A2A2D0|A2A2D0_HUMAN
tr|J3KPM9|J3KPM9_HUMAN
sp|P26447|S10A4_HUMAN
sp|A2RTX5|SYTC2_HUMAN
tr|B2R5W3|B2R5W3_HUMAN
sp|Q9NWV4|CA123_HUMAN
sp|P27348|1433T_HUMAN
sp|P02747|C1QC_HUMAN
sp|P23396|RS3_HUMAN
tr|B4DKJ4|B4DKJ4_HUMAN
tr|E5KN59|E5KN59_HUMAN
sp|O15511|ARPC5_HUMAN
tr|H3BPE7|H3BPE7_HUMAN
tr|B4DEA6|B4DEA6_HUMAN
tr|Q7Z5G3|Q7Z5G3_HUMAN
tr|B3KN06|B3KN06_HUMAN
tr|B4DL49|B4DL49_HUMAN
tr|Q53Y47|Q53Y47_HUMAN
sp|P07360|CO8G_HUMAN
tr|B4E200|B4E200_HUMAN
tr|Q7KZA3|Q7KZA3_HUMAN
tr|B5BU25|B5BU25_HUMAN
tr|Q5U043|Q5U043_HUMAN
tr|C9J8S2|C9J8S2_HUMAN
tr|B4E3A8|B4E3A8_HUMAN
tr|A0A024R172|A0A024R172_HUMAN
sp|Q6UXB8|PI16_HUMAN
sp|Q7L311|ARMX2_HUMAN
sp|Q05707|COEA1_HUMAN
tr|G3V1Y8|G3V1Y8_HUMAN
sp|P04040|CATA_HUMAN
tr|C9J712|C9J712_HUMAN
sp|Q86TP1|PRUNE_HUMAN
sp|P05787|K2C8_HUMAN
tr|K7ERI9|K7ERI9_HUMAN
sp|O95393|BMP10_HUMAN
tr|H3BN14|H3BN14_HUMAN
tr|B4E2J1|B4E2J1_HUMAN
tr|B2RB32|B2RB32_HUMAN
tr|A0A024R2V1|A0A024R2V1_HUMAN
sp|Q9BU61|NDUF3_HUMAN
sp|Q6UWY5|OLFL1_HUMAN
sp|P23141|EST1_HUMAN
tr|B4DDB3|B4DDB3_HUMAN
tr|B3KSZ1|B3KSZ1_HUMAN
tr|B2R773|B2R773_HUMAN
tr|A0A087X0E2|A0A087X0E2_HUMAN
tr|Q5T190|Q5T190_HUMAN
sp|P52179|MYOM1_HUMAN
tr|K7ER74|K7ER74_HUMAN
tr|B4DXZ6|B4DXZ6_HUMAN
tr|A0A024R2V4|A0A024R2V4_HUMAN
tr|V9GYG9|V9GYG9_HUMAN
tr|A0A087WUF6|A0A087WUF6_HUMAN
tr|B3VL28|B3VL28_HUMAN
sp|P09471|GNAO_HUMAN
sp|Q9NVI7|ATD3A_HUMAN
tr|H3BM67|H3BM67_HUMAN
sp|P02042|HBD_HUMAN
tr|D3DXF2|D3DXF2_HUMAN
sp|P26678|PPLA_HUMAN
tr|A0A087WXX9|A0A087WXX9_HUMAN
tr|B4DX19|B4DX19_HUMAN
tr|A0A024R4D5|A0A024R4D5_HUMAN
tr|B4DMR6|B4DMR6_HUMAN
tr|H3BUX2|H3BUX2_HUMAN
tr|A0A087WTK8|A0A087WTK8_HUMAN
sp|Q9H2M9|RBGPR_HUMAN
sp|Q9Y623|MYH4_HUMAN

sp|P35579|MYH9_HUMAN
sp|P01034|CYTC_HUMAN
tr|A0A024QZX5|A0A024QZX5_HUMAN
sp|P0C0L5|CO4B_HUMAN
tr|J3QRS3|J3QRS3_HUMAN
sp|P00338|LDHA_HUMAN
sp|P14550|AK1A1_HUMAN
tr|B3KTA3|B3KTA3_HUMAN
tr|D6RGG3|D6RGG3_HUMAN
sp|P51911|CNN1_HUMAN
sp|P08294|SODE_HUMAN
tr|B4DI69|B4DI69_HUMAN
tr|B2RAY1|B2RAY1_HUMAN
sp|O43776|SYNC_HUMAN
sp|P61158|ARP3_HUMAN
sp|P01903|DRA_HUMAN
sp|Q0ZGT2|NEXN_HUMAN
sp|Q9Y5S9|RBM8A_HUMAN
tr|B4DV28|B4DV28_HUMAN
tr|Q5U071|Q5U071_HUMAN
tr|B4E2A3|B4E2A3_HUMAN
tr|A0A087WSV8|A0A087WSV8_HUMAN
tr|H3BRU6|H3BRU6_HUMAN
tr|A0A024QZJ6|A0A024QZJ6_HUMAN
sp|P0C0S5|H2AZ_HUMAN
tr|B2R6C0|B2R6C0_HUMAN
tr|Q53FI7|Q53FI7_HUMAN
sp|Q9NR28|DBLOH_HUMAN
tr|B3KSI7|B3KSI7_HUMAN
tr|Q8TCF0|Q8TCF0_HUMAN
tr|A0A087WXL8|A0A087WXL8_HUMAN
tr|G3V1V0|G3V1V0_HUMAN
sp|P26038|MOES_HUMAN
sp|P29218|IMPA1_HUMAN
sp|O75339|CILP1_HUMAN
tr|A0A024RDA6|A0A024RDA6_HUMAN
sp|P48061|SDF1_HUMAN
sp|Q9NZN3|EHD3_HUMAN
tr|A0A087X1Z3|A0A087X1Z3_HUMAN
sp|P32418|NAC1_HUMAN
tr|B3KR50|B3KR50_HUMAN
sp|P50461|CSRP3_HUMAN
tr|A0A0A0MSQ0|A0A0A0MSQ0_HUMAN
sp|P20962|PTMS_HUMAN
tr|Q5VY30|Q5VY30_HUMAN
sp|O00264|PGRC1_HUMAN
sp|Q9HCB6|SPON1_HUMAN
sp|P21397|AOFA_HUMAN
tr|S6BAR0|S6BAR0_HUMAN
tr|B4DGU4|B4DGU4_HUMAN
tr|B4DFP1|B4DFP1_HUMAN
tr|B4DVE1|B4DVE1_HUMAN
tr|U3KPS2|U3KPS2_HUMAN
tr|A0A024R9B9|A0A024R9B9_HUMAN
sp|A2RUH7|MBPHL_HUMAN
tr|A5PLL0|A5PLL0_HUMAN
tr|B3KUR3|B3KUR3_HUMAN
sp|P54652|HSP72_HUMAN
tr|A0A087WZE4|A0A087WZE4_HUMAN
sp|P02768|ALBU_HUMAN
sp|Q9H7C9|AAMDC_HUMAN
sp|Q9HBL7|PLRKT_HUMAN
sp|Q07507|DERM_HUMAN
sp|O00483|NDUA4_HUMAN
tr|B4DZL5|B4DZL5_HUMAN
tr|A0A024R1E2|A0A024R1E2_HUMAN
sp|P08729|K2C7_HUMAN
tr|A0A087X130|A0A087X130_HUMAN
sp|Q9Y676|RT18B_HUMAN
sp|Q9UJS0|CMC2_HUMAN
tr|B4DUL3|B4DUL3_HUMAN
tr|V9HW62|V9HW62_HUMAN
tr|B2R8P6|B2R8P6_HUMAN
sp|Q2TAY7|SMU1_HUMAN
sp|P00918|CAH2_HUMAN
tr|Q5IWS5|Q5IWS5_HUMAN
tr|A0A024R825|A0A024R825_HUMAN
tr|A0A024R1X8|A0A024R1X8_HUMAN
sp|Q9BX97|PLVAP_HUMAN
tr|A2RTY6|A2RTY6_HUMAN
tr|A0A087X1J7|A0A087X1J7_HUMAN
tr|A0A0A0MSG2|A0A0A0MSG2_HUMAN
tr|B3KM97|B3KM97_HUMAN
sp|P68133|ACTS_HUMAN

tr|A0A087WYR3|A0A087WYR3_HUMAN
tr|A0A024RAM4|A0A024RAM4_HUMAN
sp|O75368|SH3L1_HUMAN
tr|B4DWC4|B4DWC4_HUMAN
tr|Q15374|Q15374_HUMAN
sp|P06727|APOA4_HUMAN
sp|P19021|AMD_HUMAN
tr|B5BUI8|B5BUI8_HUMAN
tr|B2RDY9|B2RDY9_HUMAN
tr|A0A024RDB4|A0A024RDB4_HUMAN
tr|K7EMY7|K7EMY7_HUMAN
sp|P61970|NTF2_HUMAN
tr|J3KR44|J3KR44_HUMAN
sp|P07237|PDIA1_HUMAN
tr|A0A024R895|A0A024R895_HUMAN
tr|B3KM95|B3KM95_HUMAN
sp|P31946|1433B_HUMAN
tr|Q6N093|Q6N093_HUMAN
tr|E9PIM6|E9PIM6_HUMAN
sp|P04080|CYTB_HUMAN
tr|A0A087WUI4|A0A087WUI4_HUMAN
sp|P68036|UB2L3_HUMAN
tr|A0A087WUS0|A0A087WUS0_HUMAN
tr|J3KTF8|J3KTF8_HUMAN
tr|A9LSU1|A9LSU1_HUMAN
sp|P50897|PPT1_HUMAN
tr|M0QXK2|M0QXK2_HUMAN
sp|P00488|F13A_HUMAN
sp|O95631|NET1_HUMAN
tr|A6XNE2|A6XNE2_HUMAN
sp|Q02252|MMSA_HUMAN
sp|P18065|IBP2_HUMAN
tr|A0A087WTH0|A0A087WTH0_HUMAN
tr|B4DVR4|B4DVR4_HUMAN
tr|Q32Q10|Q32Q10_HUMAN
tr|B3KXD1|B3KXD1_HUMAN
sp|Q96QR8|PURB_HUMAN
sp|Q15181|IPYR_HUMAN
tr|V9HW38|V9HW38_HUMAN
tr|A0A024RAF2|A0A024RAF2_HUMAN
tr|B3KRM8|B3KRM8_HUMAN
tr|A0A090N8G0|A0A090N8G0_HUMAN
tr|A8K4V6|A8K4V6_HUMAN
tr|H0YL18|H0YL18_HUMAN

sp|Q09028|RBBP4_HUMAN
tr|B3KUB8|B3KUB8_HUMAN
tr|Q53FB0|Q53FB0_HUMAN
tr|A8K4G7|A8K4G7_HUMAN
tr|X6RFL8|X6RFL8_HUMAN
tr|B2RB23|B2RB23_HUMAN
tr|B4DQ92|B4DQ92_HUMAN
sp|P62306|RUXF_HUMAN
tr|E7EVA0|E7EVA0_HUMAN
sp|P07737|PROF1_HUMAN
sp|Q04917|1433F_HUMAN
tr|A0A075B6R9|A0A075B6R9_HUMAN
sp|P62857|RS28_HUMAN
tr|A0A024R258|A0A024R258_HUMAN
tr|J3KRG9|J3KRG9_HUMAN
tr|A0A024R9E2|A0A024R9E2_HUMAN
sp|Q8N474|SFRP1_HUMAN
tr|B3KX72|B3KX72_HUMAN
tr|Q3LIE9|Q3LIE9_HUMAN
sp|P08493|MGP_HUMAN
sp|P61626|LYSC_HUMAN
sp|O15144|ARPC2_HUMAN
sp|Q01469|FABP5_HUMAN
sp|Q15435|PP1R7_HUMAN
tr|B2R6M6|B2R6M6_HUMAN
tr|Q5JNW7|Q5JNW7_HUMAN
tr|B2RDQ3|B2RDQ3_HUMAN
sp|Q969G5|PRDBP_HUMAN
sp|Q16543|CDC37_HUMAN
tr|B5BUB5|B5BUB5_HUMAN
tr|A8K3B0|A8K3B0_HUMAN
sp|P67936|TPM4_HUMAN
tr|B4DWA5|B4DWA5_HUMAN
tr|B2R4M6|B2R4M6_HUMAN
tr|Q6IBG1|Q6IBG1_HUMAN
tr|B4DNP0|B4DNP0_HUMAN
sp|Q9Y266|NUDC_HUMAN
tr|J3KS22|J3KS22_HUMAN
tr|H0YIV4|H0YIV4_HUMAN
sp|Q96JB5|CK5P3_HUMAN
sp|P02763|A1AG1_HUMAN
sp|Q6EEV6|SUMO4_HUMAN
sp|Q15293|RCN1_HUMAN
sp|P78539|SRPX_HUMAN

sp|P62820|RAB1A_HUMAN
tr|A1L172|A1L172_HUMAN
sp|P12111|CO6A3_HUMAN
tr|A0A5E4|A0A5E4_HUMAN
sp|P31150|GDIA_HUMAN
tr|C9JWC3|C9JWC3_HUMAN
sp|Q13103|SPP24_HUMAN
sp|P52907|CAZA1_HUMAN
tr|X6RLJ0|X6RLJ0_HUMAN
sp|Q15746|MYLK_HUMAN
sp|Q8WUW1|BRK1_HUMAN
tr|B7Z832|B7Z832_HUMAN
sp|Q96AG4|LRC59_HUMAN
sp|P05387|RLA2_HUMAN
tr|Q53FB6|Q53FB6_HUMAN
sp|P07305|H10_HUMAN
tr|A0A024R0K2|A0A024R0K2_HUMAN
sp|O75369|FLNB_HUMAN
sp|Q4KWH8|PLCH1_HUMAN
sp|Q702N8|XIRP1_HUMAN
tr|A0A024R884|A0A024R884_HUMAN
sp|Q15417|CNN3_HUMAN
tr|B2R6S5|B2R6S5_HUMAN
tr|B4E0U6|B4E0U6_HUMAN
tr|A0A087X1K9|A0A087X1K9_HUMAN
tr|A0A087X094|A0A087X094_HUMAN
tr|A0A024R5M3|A0A024R5M3_HUMAN
sp|Q5R372|RBG1L_HUMAN
sp|Q96D15|RCN3_HUMAN
tr|B0QYK0|B0QYK0_HUMAN
tr|A0A0A0MSV6|A0A0A0MSV6_HUMAN
sp|Q9BRA2|TXD17_HUMAN
tr|E7EQ72|E7EQ72_HUMAN
sp|P31949|S10AB_HUMAN
tr|Q6P528|Q6P528_HUMAN
tr|A0A0A0MTH3|A0A0A0MTH3_HUMAN
tr|B3KXB8|B3KXB8_HUMAN
sp|P37802|TAGL2_HUMAN
tr|Q5CZH6|Q5CZH6_HUMAN
sp|Q9UII2|ATIF1_HUMAN
tr|A0A087WZF1|A0A087WZF1_HUMAN

Chloride Channels Are Involved in the Development of Atrial Fibrillation – A Transcriptomic and proteomic Study

Yi-Yao Jiang, Hai-Tao Hou, Qin Yang, Xiao-Cheng Liu, and *Guo-Wei He

Supplementary Table 7. The differential expression between RA-SR and RA-AF

RA-SR vs RA-AF	
Up-regulation	Down-regulation
tr|M0QXF9|M0QXF9_HUMAN	sp|P27338|AOFB_HUMAN
sp|P31146|COR1A_HUMAN	sp|P08727|K1C19_HUMAN
sp|Q07960|RHG01_HUMAN	tr|B4DVZ0|B4DVZ0_HUMAN
sp|Q15942|ZYX_HUMAN	tr|B4DNG0|B4DNG0_HUMAN
sp|Q9NR12|PDLI7_HUMAN	sp|P23142|FBLN1_HUMAN
tr|J3KQ99|J3KQ99_HUMAN	sp|Q8WVV4|POF1B_HUMAN
sp|P12110|CO6A2_HUMAN	sp|Q9NZ08|ERAP1_HUMAN
sp|Q8WW22|DNJA4_HUMAN	sp|O95210|STBD1_HUMAN
sp|Q9UMX5|NENF_HUMAN	sp|Q13228|SBP1_HUMAN
tr|B2R548|B2R548_HUMAN	tr|M0QYS1|M0QYS1_HUMAN
sp|P63104|1433Z_HUMAN	sp|P21912|SDHB_HUMAN
sp|P08311|CATG_HUMAN	tr|H0YI09|H0YI09_HUMAN
tr|H0Y5J4|H0Y5J4_HUMAN	sp|P43652|AFAM_HUMAN
tr|A0A087WUQ6|A0A087WUQ6_HUMAN	sp|P30043|BLVRB_HUMAN
sp|Q9BUF5|TBB6_HUMAN	tr|A0A024R172|A0A024R172_HUMAN
sp|Q9H479|FN3K_HUMAN	tr|F8W1A4|F8W1A4_HUMAN
sp|Q9NP98|MYOZ1_HUMAN	tr|H3BNX8|H3BNX8_HUMAN
tr|B7Z6G4|B7Z6G4_HUMAN	tr|Q53FC3|Q53FC3_HUMAN
tr|A4UCS6|A4UCS6_HUMAN	sp|P04040|CATA_HUMAN
tr|H0YGX7|H0YGX7_HUMAN	tr|A0A087WT47|A0A087WT47_HUMAN
tr|G3V3X5|G3V3X5_HUMAN	tr|K7ERI9|K7ERI9_HUMAN
sp|Q9BX66|SRBS1_HUMAN	tr|Q6ZN40|Q6ZN40_HUMAN
sp|P19652|A1AG2_HUMAN	tr|H3BN14|H3BN14_HUMAN
tr|Q6FIE5|Q6FIE5_HUMAN	tr|B4E2J1|B4E2J1_HUMAN
sp|Q9NZA1|CLIC5_HUMAN	sp|Q4G0N4|NAKD2_HUMAN
sp|P13929|ENOB_HUMAN	sp|P23141|EST1_HUMAN
tr|A0A024R493|A0A024R493_HUMAN	sp|P15090|FABP4_HUMAN
tr|K7EKW4|K7EKW4_HUMAN	tr|B4DVV3|B4DVV3_HUMAN
sp|P01616|KV203_HUMAN	tr|M0R0P7|M0R0P7_HUMAN
sp|P07437|TBB5_HUMAN	sp|A2RTX5|SYTC2_HUMAN
tr|Q9UL89|Q9UL89_HUMAN	sp|P23368|MAOM_HUMAN
tr|H0Y614|H0Y614_HUMAN	sp|P14927|QCR7_HUMAN
tr|A0A024R091|A0A024R091_HUMAN	tr|Q0EFC6|Q0EFC6_HUMAN
tr|Q5RKT7|Q5RKT7_HUMAN	sp|P02042|HBD_HUMAN

tr|H7BZJ3|H7BZJ3_HUMAN
tr|E7EMG9|E7EMG9_HUMAN
tr|W6A4U0|W6A4U0_HUMAN
tr|B4DEX8|B4DEX8_HUMAN
tr|B4DUJ6|B4DUJ6_HUMAN
tr|A2A2D0|A2A2D0_HUMAN
tr|A0A068LKR7|A0A068LKR7_HUMAN
sp|O95817|BAG3_HUMAN
tr|H3BPK3|H3BPK3_HUMAN
sp|P27348|1433T_HUMAN
tr|B4DKJ4|B4DKJ4_HUMAN
tr|Q5U000|Q5U000_HUMAN
tr|E5KN59|E5KN59_HUMAN
sp|Q9UDY4|DNJB4_HUMAN
tr|B4DS05|B4DS05_HUMAN
tr|B4DEA6|B4DEA6_HUMAN
tr|B4DL49|B4DL49_HUMAN
tr|Q53Y47|Q53Y47_HUMAN
tr|A0A024R6X1|A0A024R6X1_HUMAN
tr|Q5U043|Q5U043_HUMAN
sp|P35754|GLRX1_HUMAN
tr|B4E3A8|B4E3A8_HUMAN
sp|P35579|MYH9_HUMAN
tr|A0A024QZX5|A0A024QZX5_HUMAN
tr|A0A024QZT9|A0A024QZT9_HUMAN
sp|P0C0L5|CO4B_HUMAN
tr|A0A0A0MQY0|A0A0A0MQY0_HUMAN
tr|B2R6D7|B2R6D7_HUMAN
tr|E9PB61|E9PB61_HUMAN
sp|P51911|CNN1_HUMAN
tr|B4DI69|B4DI69_HUMAN
tr|B2RAY1|B2RAY1_HUMAN
sp|Q9H987|SYP2L_HUMAN
sp|Q8TAE6|PP14C_HUMAN
tr|B5BTY4|B5BTY4_HUMAN
sp|P61158|ARP3_HUMAN
sp|P01903|DRA_HUMAN
sp|Q0ZGT2|NEXN_HUMAN
tr|B4DV28|B4DV28_HUMAN
tr|H3BRN4|H3BRN4_HUMAN
tr|B3KSI7|B3KSI7_HUMAN
tr|Q8TCF0|Q8TCF0_HUMAN
tr|G3V1V0|G3V1V0_HUMAN
sp|P26038|MOES_HUMAN
sp|O75947|ATP5H_HUMAN
tr|B7Z4V9|B7Z4V9_HUMAN
sp|Q14008|CKAP5_HUMAN
tr|A0A024R4D5|A0A024R4D5_HUMAN
sp|P48047|ATPO_HUMAN
tr|Q9NSD0|Q9NSD0_HUMAN
sp|Q8N5K1|CISD2_HUMAN
tr|Q5VY30|Q5VY30_HUMAN
sp|P14555|PA2GA_HUMAN
tr|D3DVH1|D3DVH1_HUMAN
sp|P09669|COX6C_HUMAN
tr|K7ESP4|K7ESP4_HUMAN
sp|Q9NY47|CA2D2_HUMAN
tr|B1AP13|B1AP13_HUMAN
sp|P62760|VISL1_HUMAN
tr|E9PN17|E9PN17_HUMAN
tr|A0A024R9B9|A0A024R9B9_HUMAN
tr|B3KUR3|B3KUR3_HUMAN
tr|A8JZZ8|A8JZZ8_HUMAN
tr|A0A087WZE4|A0A087WZE4_HUMAN
sp|Q5VTU8|AT5EL_HUMAN
tr|I1VZV6|I1VZV6_HUMAN
tr|E7EQV9|E7EQV9_HUMAN
tr|G3V325|G3V325_HUMAN
tr|B2R6C0|B2R6C0_HUMAN
sp|P00918|CAH2_HUMAN
sp|Q96FQ6|S10AG_HUMAN
tr|A0A024R825|A0A024R825_HUMAN
sp|Q9BS92|NPS3B_HUMAN
sp|Q9UBG0|MRC2_HUMAN
tr|B7Z5J1|B7Z5J1_HUMAN
sp|P16157|ANK1_HUMAN
tr|D3GKD8|D3GKD8_HUMAN
tr|A8K9B2|A8K9B2_HUMAN
sp|Q4G176|ACSF3_HUMAN
tr|Q8N7G1|Q8N7G1_HUMAN
tr|A0A0A0MTQ5|A0A0A0MTQ5_HUMAN
tr|A0A0A0MR33|A0A0A0MR33_HUMAN
sp|Q9NPJ3|ACO13_HUMAN
tr|H0YL90|H0YL90_HUMAN
tr|B4DMD3|B4DMD3_HUMAN
tr|B2R6F7|B2R6F7_HUMAN
sp|Q13057|COASY_HUMAN
sp|P05154|IPSP_HUMAN

tr|G3V4P8|G3V4P8_HUMAN
tr|A0A024RDA6|A0A024RDA6_HUMAN
sp|Q9NZN3|EHD3_HUMAN
tr|D6RAW2|D6RAW2_HUMAN
tr|Q5STX8|Q5STX8_HUMAN
sp|Q9GZM7|TINAL_HUMAN
sp|Q13885|TBB2A_HUMAN
sp|Q01995|TAGL_HUMAN
sp|P50461|CSRP3_HUMAN
tr|A0A0A0MSQ0|A0A0A0MSQ0_HUMAN
sp|P20962|PTMS_HUMAN
tr|A0A087WYR3|A0A087WYR3_HUMAN
tr|B4DWC4|B4DWC4_HUMAN
tr|B3KQK4|B3KQK4_HUMAN
tr|Q15374|Q15374_HUMAN
sp|P06727|APOA4_HUMAN
tr|B5BUI8|B5BUI8_HUMAN
tr|B2RDY9|B2RDY9_HUMAN
sp|O60237|MYPT2_HUMAN
tr|Q59GM9|Q59GM9_HUMAN
sp|Q9BXX0|EMIL2_HUMAN
sp|P19022|CADH2_HUMAN
tr|A0A024R895|A0A024R895_HUMAN
tr|B3KXC3|B3KXC3_HUMAN
sp|P31946|1433B_HUMAN
tr|Q6N093|Q6N093_HUMAN
tr|A0A087WUI4|A0A087WUI4_HUMAN
sp|P04080|CYTB_HUMAN
sp|P02671|FIBA_HUMAN
tr|J3KTF8|J3KTF8_HUMAN
tr|B4DH02|B4DH02_HUMAN
sp|Q92890|UFD1_HUMAN
sp|P00488|F13A_HUMAN
sp|P35221|CTNA1_HUMAN
sp|Q02252|MMSA_HUMAN
sp|P18065|IBP2_HUMAN
tr|A0A087WTH0|A0A087WTH0_HUMAN
tr|B3KXD1|B3KXD1_HUMAN
tr|V9HW38|V9HW38_HUMAN
tr|A0A024RAF2|A0A024RAF2_HUMAN
sp|P01619|KV301_HUMAN
tr|A0A090N8G0|A0A090N8G0_HUMAN
sp|Q16082|HSPB2_HUMAN
sp|O95393|BMP10_HUMAN
tr|A0A024RDX4|A0A024RDX4_HUMAN
tr|D3DWJ7|D3DWJ7_HUMAN
sp|P05787|K2C8_HUMAN
sp|Q9Y230|RUVB2_HUMAN
tr|A0A024RBE8|A0A024RBE8_HUMAN
sp|P41222|PTGDS_HUMAN
sp|P82673|RT35_HUMAN
tr|B2RA03|B2RA03_HUMAN
tr|B2RB32|B2RB32_HUMAN
tr|B3KUE5|B3KUE5_HUMAN
tr|A0A024R2V1|A0A024R2V1_HUMAN
tr|I3L0E3|I3L0E3_HUMAN
sp|Q6UWY5|OLFL1_HUMAN
tr|Q7Z3Z1|Q7Z3Z1_HUMAN
tr|Q5T8U5|Q5T8U5_HUMAN
tr|B3KSZ1|B3KSZ1_HUMAN
sp|Q13425|SNTB2_HUMAN
sp|P61626|LYSC_HUMAN
tr|A0A087WUF6|A0A087WUF6_HUMAN
sp|Q8WW59|SPRY4_HUMAN
tr|H0YAC1|H0YAC1_HUMAN
sp|O60240|PLIN1_HUMAN
tr|B4DX19|B4DX19_HUMAN
sp|P26678|PPLA_HUMAN
tr|B7Z601|B7Z601_HUMAN
sp|P12429|ANXA3_HUMAN
tr|B4DMR6|B4DMR6_HUMAN
sp|Q5T481|RBM20_HUMAN
tr|H3BUX2|H3BUX2_HUMAN
tr|A0A024R4Y7|A0A024R4Y7_HUMAN
sp|O00264|PGRC1_HUMAN
sp|P21397|AOFA_HUMAN
sp|P55001|MFAP2_HUMAN
tr|Q2TB59|Q2TB59_HUMAN
tr|B4DVE1|B4DVE1_HUMAN
tr|A0A059U7G5|A0A059U7G5_HUMAN
tr|B2RDE0|B2RDE0_HUMAN
tr|A0A0A0MRE3|A0A0A0MRE3_HUMAN
sp|A2RUH7|MBPHL_HUMAN
tr|H0YHA7|H0YHA7_HUMAN
sp|Q9H7C9|AAMDC_HUMAN
tr|A0A087WT59|A0A087WT59_HUMAN
sp|Q07507|DERM_HUMAN
sp|Q96IX5|USMG5_HUMAN

tr|Q53FB0|Q53FB0_HUMAN
tr|C9JC84|C9JC84_HUMAN
sp|Q15847|ADIRF_HUMAN
tr|E7EVA0|E7EVA0_HUMAN
sp|P07737|PROF1_HUMAN
sp|Q04917|1433F_HUMAN
tr|A0A075B6R9|A0A075B6R9_HUMAN
sp|Q14315|FLNC_HUMAN
tr|J3KRG9|J3KRG9_HUMAN
sp|O15144|ARPC2_HUMAN
sp|Q01469|FABP5_HUMAN
sp|Q15435|PP1R7_HUMAN
tr|B2R6M6|B2R6M6_HUMAN
sp|Q9HCH3|CPNE5_HUMAN
sp|P09936|UCHL1_HUMAN
tr|A8K3B0|A8K3B0_HUMAN
sp|P67936|TPM4_HUMAN
tr|B2R4M6|B2R4M6_HUMAN
tr|H0YIV4|H0YIV4_HUMAN
sp|Q96JB5|CK5P3_HUMAN
tr|E9PGC8|E9PGC8_HUMAN
tr|Q4G1C4|Q4G1C4_HUMAN
sp|Q13442|HAP28_HUMAN
sp|P02763|A1AG1_HUMAN
sp|Q14894|CRYM_HUMAN
sp|Q6EEV6|SUMO4_HUMAN
sp|P78539|SRPX_HUMAN
tr|Q59EG0|Q59EG0_HUMAN
sp|P62820|RAB1A_HUMAN
sp|P19623|SPEE_HUMAN
tr|A1L172|A1L172_HUMAN
sp|P31150|GDIA_HUMAN
tr|A0A5E4|A0A5E4_HUMAN
sp|Q13103|SPP24_HUMAN
tr|C9JWC3|C9JWC3_HUMAN
sp|Q15746|MYLK_HUMAN
sp|Q9BW30|TPPP3_HUMAN
sp|Q9UHL4|DPP2_HUMAN
tr|Q3B874|Q3B874_HUMAN
tr|Q53FB6|Q53FB6_HUMAN
sp|P07305|H10_HUMAN
tr|F8WAR2|F8WAR2_HUMAN
sp|Q9UJY1|HSPB8_HUMAN
sp|Q4KWH8|PLCH1_HUMAN
tr|A0A024R248|A0A024R248_HUMAN
tr|I3L2B0|I3L2B0_HUMAN
sp|O00483|NDUA4_HUMAN
tr|B4DZL5|B4DZL5_HUMAN
tr|H0YBX2|H0YBX2_HUMAN
tr|A0A087X130|A0A087X130_HUMAN
sp|P08729|K2C7_HUMAN
tr|B2R8P6|B2R8P6_HUMAN
sp|P14854|CX6B1_HUMAN
tr|A0A0A0MTR1|A0A0A0MTR1_HUMAN
sp|Q2TAY7|SMU1_HUMAN
tr|Q5IWS5|Q5IWS5_HUMAN
tr|E5RHP7|E5RHP7_HUMAN
sp|P09455|RET1_HUMAN
tr|H7BXZ6|H7BXZ6_HUMAN
sp|P25325|THTM_HUMAN
sp|Q9BX97|PLVAP_HUMAN
sp|P68133|ACTS_HUMAN

sp|P09211|GSTP1_HUMAN
sp|Q702N8|XIRP1_HUMAN
sp|O94811|TPPP_HUMAN
tr|A0A024R884|A0A024R884_HUMAN
sp|P12109|CO6A1_HUMAN
sp|Q15417|CNN3_HUMAN
tr|B2R6S5|B2R6S5_HUMAN
sp|P37837|TALDO_HUMAN
sp|O15061|SYNEM_HUMAN
sp|P15848|ARSB_HUMAN
sp|Q15843|NEDD8_HUMAN
tr|H0Y7A7|H0Y7A7_HUMAN
tr|Q6P528|Q6P528_HUMAN
sp|Q9UBY9|HSPB7_HUMAN
tr|B3KXB8|B3KXB8_HUMAN
tr|A0A0A0MSG2|A0A0A0MSG2_HUMAN
sp|P37802|TAGL2_HUMAN
tr|A0A087WZF1|A0A087WZF1_HUMAN

Chloride Channels Are Involved in the Development of Atrial Fibrillation – A Transcriptomic and proteomic Study

Yi-Yao Jiang, Hai-Tao Hou, Qin Yang, Xiao-Cheng Liu, and *Guo-Wei He

Supplementary Table 8. The differential expression between RA-AF and LA-AF

RA-AF vs LA-AF	
Up-regulation	Down-regulation
tr|A0A0A0MSZ4|A0A0A0MSZ4_HUMAN	sp|O60237|MYPT2_HUMAN
sp|P31146|COR1A_HUMAN	tr|A5YM48|A5YM48_HUMAN
tr|A8K1D2|A8K1D2_HUMAN	sp|P02792|FRIL_HUMAN
tr|A0A0A0MSQ0|A0A0A0MSQ0_HUMAN	tr|Q5UGI6|Q5UGI6_HUMAN
sp|P05109|S10A8_HUMAN	sp|Q5BKX8|MURC_HUMAN
tr|E9PLM6|E9PLM6_HUMAN	sp|P19022|CADH2_HUMAN
tr|B4DVZ0|B4DVZ0_HUMAN	sp|Q9NQC3|RTN4_HUMAN
sp|Q9NR12|PDLI7_HUMAN	tr|B4DL07|B4DL07_HUMAN
tr|Q6IB58|Q6IB58_HUMAN	tr|S6AWE6|S6AWE6_HUMAN
tr|B2R941|B2R941_HUMAN	tr|H0Y9H2|H0Y9H2_HUMAN
sp|P49327|FAS_HUMAN	sp|A4UGR9|XIRP2_HUMAN
sp|O43707|ACTN4_HUMAN	sp|Q6ZP80|TM182_HUMAN
sp|P06899|H2B1J_HUMAN	sp|Q9NP98|MYOZ1_HUMAN
tr|A0A024QZ34|A0A024QZ34_HUMAN	sp|P35221|CTNA1_HUMAN
tr|B2ZGL7|B2ZGL7_HUMAN	tr|A4UCS6|A4UCS6_HUMAN
tr|B3KTQ2|B3KTQ2_HUMAN	sp|Q99959|PKP2_HUMAN
sp|Q03135|CAV1_HUMAN	tr|G3V1Y8|G3V1Y8_HUMAN
tr|Q0D2M2|Q0D2M2_HUMAN	sp|P01619|KV301_HUMAN
tr|H0YMF4|H0YMF4_HUMAN	sp|Q86TP1|PRUNE_HUMAN
tr|B3KRN4|B3KRN4_HUMAN	sp|O95393|BMP10_HUMAN
tr|A1KY36|A1KY36_HUMAN	tr|A0A075B6R9|A0A075B6R9_HUMAN
tr|A0A024R035|A0A024R035_HUMAN	tr|A0N5G5|A0N5G5_HUMAN
tr|E9PIM6|E9PIM6_HUMAN	tr|A8K141|A8K141_HUMAN
tr|A9LSU1|A9LSU1_HUMAN	tr|H0UI60|H0UI60_HUMAN
sp|O95631|NET1_HUMAN	tr|K7ER74|K7ER74_HUMAN
tr|A6XNE2|A6XNE2_HUMAN	tr|V9GYG9|V9GYG9_HUMAN
tr|H0YGX7|H0YGX7_HUMAN	tr|A0A0A0MS87|A0A0A0MS87_HUMAN
tr|B2RA94|B2RA94_HUMAN	sp|P09936|UCHL1_HUMAN
tr|B3KVC9|B3KVC9_HUMAN	sp|Q96DG6|CMBL_HUMAN
tr|B4DTY8|B4DTY8_HUMAN	tr|A0A087WTE4|A0A087WTE4_HUMAN
sp|P15121|ALDR_HUMAN	tr|B4DX19|B4DX19_HUMAN
tr|E2RVJ0|E2RVJ0_HUMAN	tr|Q5T9B9|Q5T9B9_HUMAN
sp|P05787|K2C8_HUMAN	tr|H0YIV4|H0YIV4_HUMAN
tr|Q6ZN40|Q6ZN40_HUMAN	tr|E9PGC8|E9PGC8_HUMAN
tr|B3KUB8|B3KUB8_HUMAN	tr|B4DGU4|B4DGU4_HUMAN

tr|H3BN14|H3BN14_HUMAN
sp|O76076|WISP2_HUMAN
sp|P10915|HPLN1_HUMAN
tr|B2RA03|B2RA03_HUMAN
tr|Q59FG9|Q59FG9_HUMAN
sp|Q02338|BDH_HUMAN
sp|P59665|DEF1_HUMAN
sp|Q8N474|SFRP1_HUMAN
tr|F2Z3A7|F2Z3A7_HUMAN
tr|H0YG03|H0YG03_HUMAN
sp|Q9NZM1|MYOF_HUMAN
sp|P08493|MGP_HUMAN
tr|J3QRN6|J3QRN6_HUMAN
sp|P61626|LYSC_HUMAN
sp|P15090|FABP4_HUMAN
sp|Q01469|FABP5_HUMAN
tr|A0A087WU72|A0A087WU72_HUMAN
sp|A2RTX5|SYTC2_HUMAN
tr|B2R5W3|B2R5W3_HUMAN
sp|Q969G5|PRDBP_HUMAN
sp|P02042|HBD_HUMAN
sp|Q9NZN4|EHD2_HUMAN
sp|P02747|C1QC_HUMAN
sp|P23396|RS3_HUMAN
sp|O60240|PLIN1_HUMAN
tr|B4DWA5|B4DWA5_HUMAN
tr|B2R4M6|B2R4M6_HUMAN
tr|A0A024QZE7|A0A024QZE7_HUMAN
sp|P12429|ANXA3_HUMAN
sp|Q8NBX0|SCPDL_HUMAN
tr|J3KS22|J3KS22_HUMAN
tr|H9E7F4|H9E7F4_HUMAN
tr|D3DVH1|D3DVH1_HUMAN
sp|P09669|COX6C_HUMAN
sp|Q96CN7|ISOC1_HUMAN
tr|B4E200|B4E200_HUMAN
sp|Q92506|DHB8_HUMAN
sp|P08572|CO4A2_HUMAN
tr|J3QRS3|J3QRS3_HUMAN
sp|P02743|SAMP_HUMAN
tr|X6RLJ0|X6RLJ0_HUMAN
sp|A2RUH7|MBPHL_HUMAN
sp|Q15746|MYLK_HUMAN
tr|B7Z832|B7Z832_HUMAN
tr|B4DFP1|B4DFP1_HUMAN
tr|A0A0A0MQY0|A0A0A0MQY0_HUMAN
tr|K7EMC7|K7EMC7_HUMAN
tr|C9JWC3|C9JWC3_HUMAN
tr|A5PLL0|A5PLL0_HUMAN
sp|P54652|HSP72_HUMAN
sp|Q9BW30|TPPP3_HUMAN
tr|A0A024RC87|A0A024RC87_HUMAN
sp|P02768|ALBU_HUMAN
tr|B1ALD9|B1ALD9_HUMAN
sp|A6NMZ7|CO6A6_HUMAN
sp|O75147|OBSL1_HUMAN
sp|Q07507|DERM_HUMAN
sp|Q9UJY1|HSPB8_HUMAN
tr|Q9HCC1|Q9HCC1_HUMAN
tr|Q53FN1|Q53FN1_HUMAN
sp|O94811|TPPP_HUMAN
sp|Q8NDY3|ARHL1_HUMAN
tr|A0A024R884|A0A024R884_HUMAN
sp|Q8TAE6|PP14C_HUMAN
sp|Q9UJS0|CMC2_HUMAN
sp|Q9Y676|RT18B_HUMAN
sp|P15848|ARSB_HUMAN
tr|H3BRN4|H3BRN4_HUMAN
tr|A0A0A0MTR1|A0A0A0MTR1_HUMAN
sp|Q9Y6I3|EPN1_HUMAN
sp|P02144|MYG_HUMAN
sp|P01023|A2MG_HUMAN
tr|Q53GU8|Q53GU8_HUMAN
sp|Q06828|FMOD_HUMAN
tr|H0Y8G4|H0Y8G4_HUMAN
tr|Q6P528|Q6P528_HUMAN
sp|Q9UBY9|HSPB7_HUMAN
tr|A0A087X1J7|A0A087X1J7_HUMAN
tr|A0A0A0MSG2|A0A0A0MSG2_HUMAN

tr|B0QYN7|B0QYN7_HUMAN
sp|P51911|CNN1_HUMAN
sp|P02649|APOE_HUMAN
tr|I1VZV6|I1VZV6_HUMAN
sp|Q96IX5|USMG5_HUMAN
tr|E7EQV9|E7EQV9_HUMAN
sp|Q8N5M1|ATPF2_HUMAN
tr|B4E380|B4E380_HUMAN
sp|Q15417|CNN3_HUMAN
sp|P13010|XRCC5_HUMAN
tr|B4DEA7|B4DEA7_HUMAN
sp|P08729|K2C7_HUMAN
tr|G3XAP6|G3XAP6_HUMAN
sp|P20700|LMNB1_HUMAN
tr|B0QYK0|B0QYK0_HUMAN
tr|A0A024QZJ6|A0A024QZJ6_HUMAN
tr|Q6LAP8|Q6LAP8_HUMAN
tr|B2R6C0|B2R6C0_HUMAN
sp|P00918|CAH2_HUMAN
tr|B4DZG7|B4DZG7_HUMAN
tr|Q5IWS5|Q5IWS5_HUMAN
tr|G3V1V0|G3V1V0_HUMAN
tr|A0A024R825|A0A024R825_HUMAN
tr|Q53GL5|Q53GL5_HUMAN
tr|E5RHP7|E5RHP7_HUMAN
sp|Q8WWP7|GIMA1_HUMAN
sp|O75339|CILP1_HUMAN
sp|Q969Z3|MARC2_HUMAN
sp|Q16853|AOC3_HUMAN
sp|P48061|SDF1_HUMAN
tr|B7Z9B8|B7Z9B8_HUMAN
sp|Q9GZM7|TINAL_HUMAN

Chloride Channels Are Involved in the Development of Atrial Fibrillation – A Transcriptomic and proteomic Study

Yi-Yao Jiang, Hai-Tao Hou, Qin Yang, Xiao-Cheng Liu, and *Guo-Wei He

Supplementary Table 9. The differential expression of CLICs in transcriptome

Gene ID	Symbol	Log2 Ratio & Probability		
		LA-AF/RA-SR	RA-AF/RA-SR	LA-AF/RA-AF
1192	CLIC1	0.19 & 0.37	0.09 & 0.22	-0.09 & 0.23
1193	CLIC2	0.11 & 0.19	0.09 & 0.17	-0.02 & 0.09
9022	CLIC3	-0.96 & 0.63	-1.07 & 0.67	-0.11 & 0.13
25932	CLIC4	0.22 & 0.41	0.22 & 0.43	0.001 & 0.07
53405	CLIC5	-0.01 & 0.06	0.37 & 0.58	0.70 & 0.61
54102	CLIC6	-0.45 & 0.48	0.25 & 0.36	0.37 & 0.54

- : down regulation.

Chloride Channels Are Involved in the Development of Atrial Fibrillation – A Transcriptomic and proteomic Study

Yi-Yao Jiang, Hai-Tao Hou, Qin Yang, Xiao-Cheng Liu, and *Guo-Wei He

Supplementary Table 10 The primer sequences and the amplification product sizes of the target genes

Genes	Primer nucleotide sequence	Product size (bp)	Efficiency
CLIC1	F: ATTCAAACCCAGCACTCAAT<br>R: GAGACACCTTCATCTTCAGCA	130	1.942
CLIC2	F: TTCTGCGTCACACCTCTTGA<br>R: AGCCTCCTGCCTCCTGTAGA	160	1.924
CLIC3	F:TGTCAAGGCGAGTGAGGAC<br>R:CGTGGTGAGGGTGAAAGGTA	103	1.901
CLIC4	F: GCCAGAGGCTAATGAAGCAC<br>R: AGTTTGGGCAGCAGGTTG	186	1.917
CLIC5	F: CAAACGCCATCTTCATCCTT<br>R: GCCAGACACCCAAGTCCATA	107	1.963
CLIC6	F: GACCCAACATCCCGAATCTA<br>R: TTCATCAGGCAGAGGGCTAT	163	1.924
GAPDH	F: AACAGCGACACCCACTCCTC<br>R: GGAGGGGAGATTCAGTGTGGT	257	1.983

Chloride Channels Are Involved in the Development of Atrial Fibrillation – A Transcriptomic and proteomic Study

Yi-Yao Jiang, Hai-Tao Hou, Qin Yang, Xiao-Cheng Liu, and *Guo-Wei He

Supplementary figure 1. a) The correlation between samples was analyzed by Pearson method. The darker color means higher correlation. b) Differentially expressed genes were figured among individual samples. c) Top 20 statistics of pathway enrichment between individuals was analyzed. The greater dot means more genes involved in this pathway. Q value represented significantly enrichment of Pathway in differentially expressed genes.

Supplementary figure 2. Focal adhesion pathway was significant in transcriptome and proteomics.

Supplementary figure 3. Protein function was categorized in biological process, cell component and molecular function. Protein percentage was shown as different color.

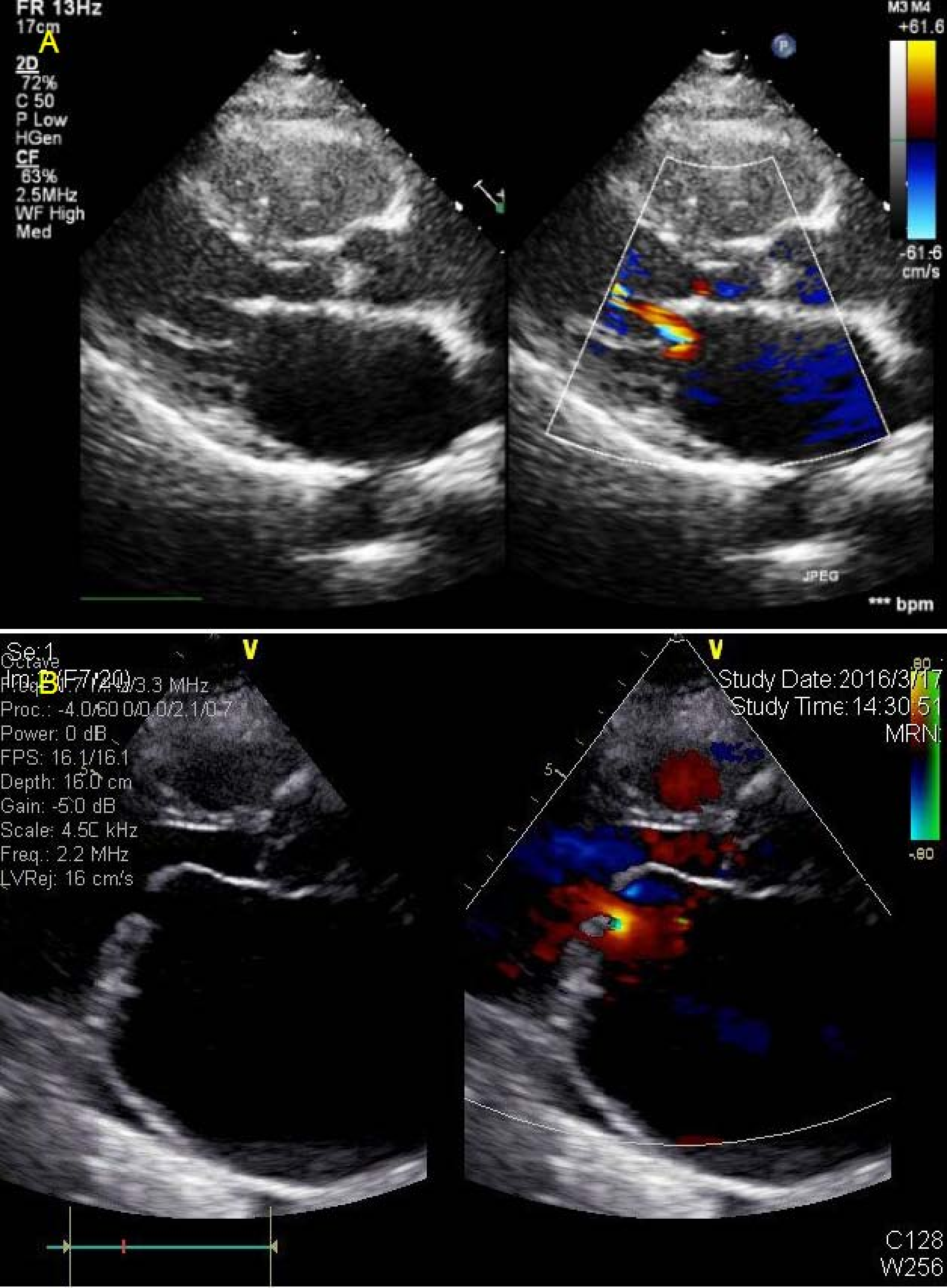
FR 13Hz
17cm
A
2D
72%
C 50
P Low
HGen
CF
63%
2.5MHz
WF High
Med
M3 M4
+61.6
-61.6
cm/s
JPEG
*** bpm
Se:1
B
Power: 0 dB
FPS: 16.1/16.1
Depth: 16.0 cm
Gain: -5.0 dB
Freq.: 2.2 MHz
LVRej: 16 cm/s
Study Date:2016/3/17
Study Time:14:30:51
MRN:
80
-80
C128
W256

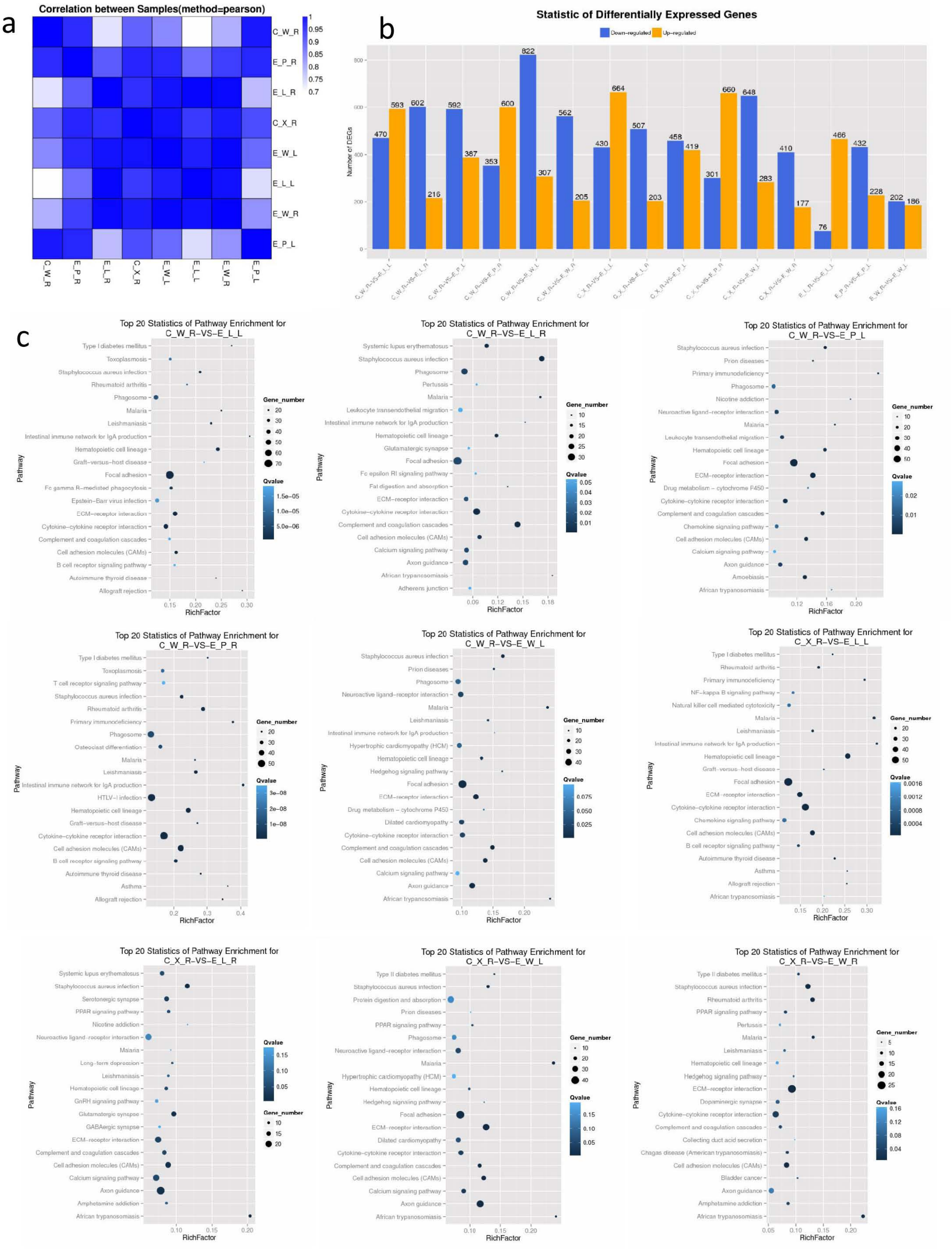

a
Correlation between Samples(method=pearson)
C_W_R
E_P_R
E_L_R
C_X_R
E_W_L
E_L_L
E_W_R
E_P_L
b
Statistic of Differentially Expressed Genes
Down-regulated
Up-regulated
Number of DEGs
c
Top 20 Statistics of Pathway Enrichment for C_W_R-VS-E_L_L
Top 20 Statistics of Pathway Enrichment for C_W_R-VS-E_L_R
Top 20 Statistics of Pathway Enrichment for C_W_R-VS-E_P_L
Top 20 Statistics of Pathway Enrichment for C_W_R-VS-E_P_R
Top 20 Statistics of Pathway Enrichment for C_W_R-VS-E_W_L
Top 20 Statistics of Pathway Enrichment for C_X_R-VS-E_L_L
Top 20 Statistics of Pathway Enrichment for C_X_R-VS-E_L_R
Top 20 Statistics of Pathway Enrichment for C_X_R-VS-E_W_L
Top 20 Statistics of Pathway Enrichment for C_X_R-VS-E_W_R
Pathway
RichFactor
Gene_number
Qvalue

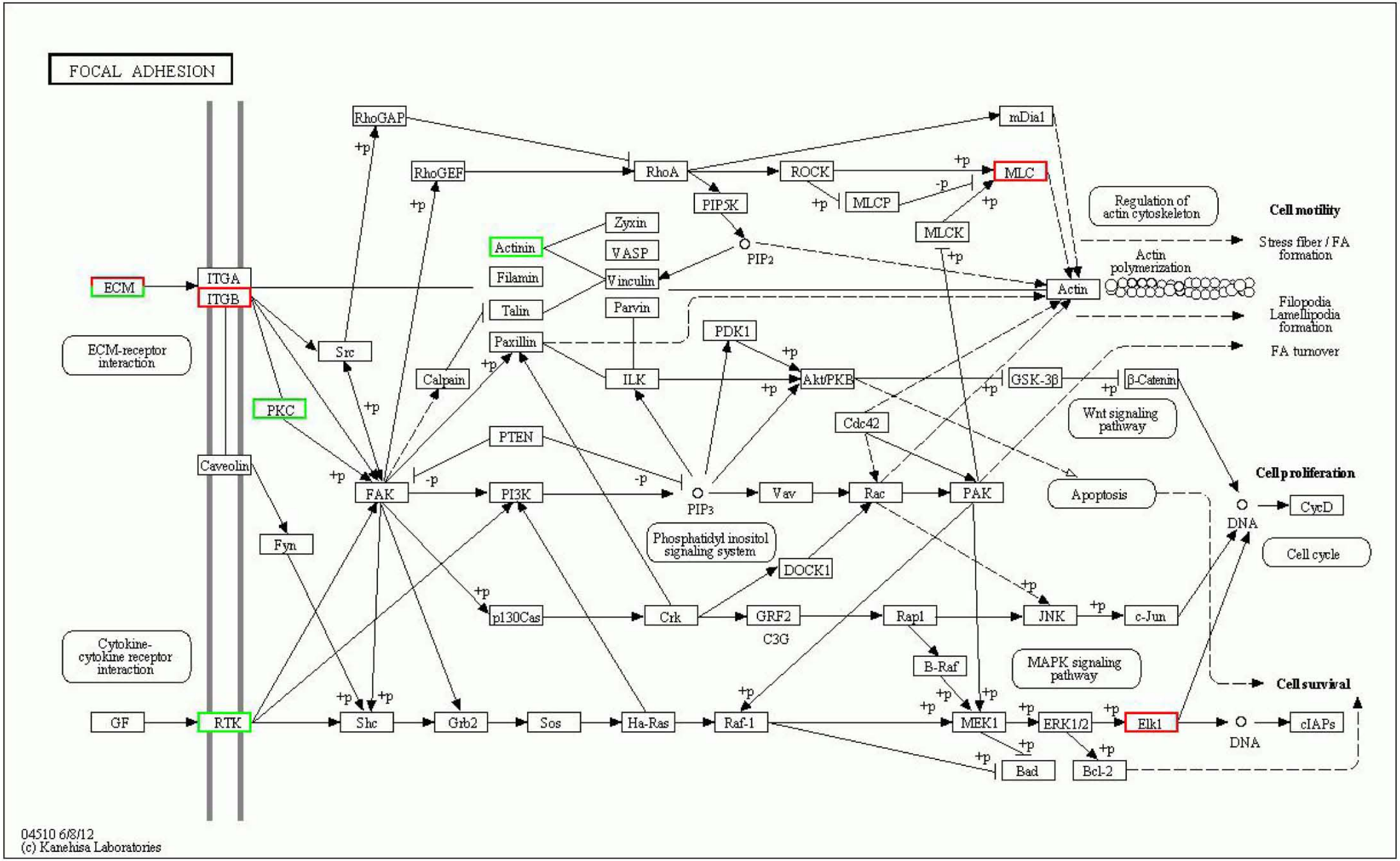
FOCAL ADHESION
RhoGAP
RhoGEF
RhoA
ROCK
MLC
mDia1
MLCP
MLCK
PIP5K
PIP2
Actinin
Zyxin
VASP
Vinculin
Filamin
Talin
Parvin
Paxillin
ECM
ITGA
ITGB
Src
Calpain
ILK
PDK1
Akt/PKB
GSK-3β
β-Catenin
PKC
PTEN
Caveolin
FAK
PI3K
PIP3
Vav
Rac
PAK
Cdc42
Apoptosis
Fyn
p130Cas
Crk
GRF2
C3G
DOCK1
Rap1
JNK
c-Jun
B-Raf
GF
RTK
Shc
Grb2
Sos
Ha-Ras
Raf-1
MEK1
ERK1/2
Elk1
Bad
Bcl-2
DNA
CycD
cIAPs
Actin
Regulation of actin cytoskeleton
Actin polymerization
Cell motility
Stress fiber / FA formation
Filopodia Lamellipodia formation
FA turnover
Wnt signaling pathway
Cell proliferation
Cell cycle
Cell survival
ECM-receptor interaction
Cytokine-cytokine receptor interaction
Phosphatidyl inositol signaling system
MAPK signaling pathway
04510 6/8/12
(c) Kanehisa Laboratories

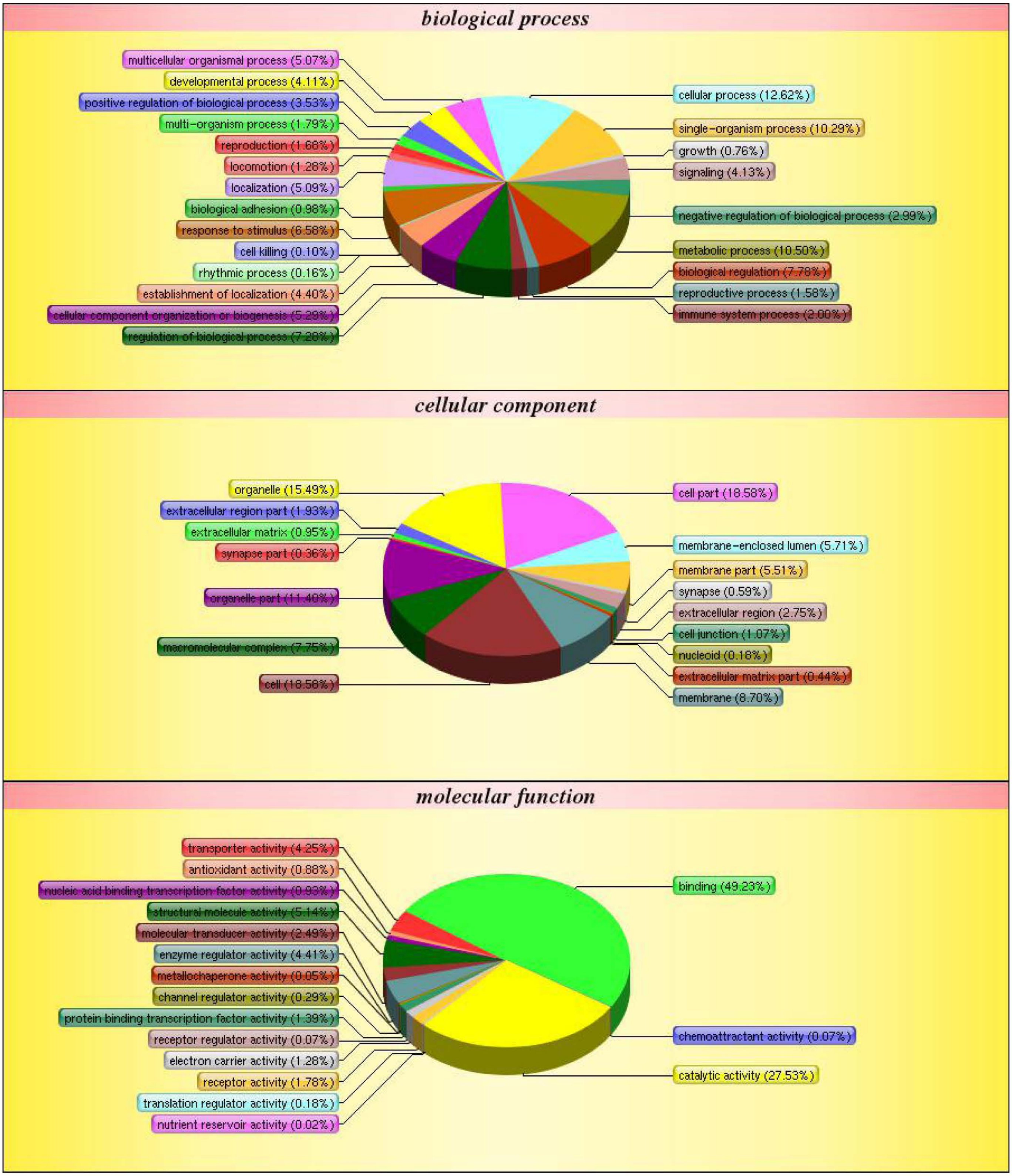
biological process
multicellular organismal process (5.07%)
developmental process (4.11%)
positive regulation of biological process (3.53%)
multi-organism process (1.79%)
reproduction (1.68%)
locomotion (1.28%)
localization (5.09%)
biological adhesion (0.98%)
response to stimulus (6.58%)
cell killing (0.10%)
rhythmic process (0.16%)
establishment of localization (4.40%)
cellular component organization or biogenesis (5.29%)
regulation of biological process (7.28%)
cellular process (12.62%)
single-organism process (10.29%)
growth (0.76%)
signaling (4.13%)
negative regulation of biological process (2.99%)
metabolic process (10.50%)
biological regulation (7.78%)
reproductive process (1.58%)
immune system process (2.00%)
cellular component
organelle (15.49%)
extracellular region part (1.93%)
extracellular matrix (0.95%)
synapse part (0.36%)
organelle part (11.40%)
macromolecular complex (7.75%)
cell (18.58%)
cell part (18.58%)
membrane-enclosed lumen (5.71%)
membrane part (5.51%)
synapse (0.59%)
extracellular region (2.75%)
cell junction (1.07%)
nucleoid (0.18%)
extracellular matrix part (0.44%)
membrane (8.70%)
molecular function
transporter activity (4.25%)
antioxidant activity (0.88%)
nucleic acid binding transcription factor activity (0.93%)
structural molecule activity (5.14%)
molecular transducer activity (2.49%)
enzyme regulator activity (4.41%)
metallochaperone activity (0.05%)
channel regulator activity (0.29%)
protein binding transcription factor activity (1.39%)
receptor regulator activity (0.07%)
electron carrier activity (1.28%)
receptor activity (1.78%)
translation regulator activity (0.18%)
nutrient reservoir activity (0.02%)
binding (49.23%)
chemoattractant activity (0.07%)
catalytic activity (27.53%)
